# The predictive power of data-processing statistics

**DOI:** 10.1107/S2052252520000895

**Published:** 2020-02-27

**Authors:** Melanie Vollmar, James M. Parkhurst, Dominic Jaques, Arnaud Baslé, Garib N. Murshudov, David G. Waterman, Gwyndaf Evans

**Affiliations:** a Diamond Light Source Ltd, Harwell Science and Innovation Campus, Didcot OX11 0DE, England; b MRC Laboratory of Molecular Biology, Francis Crick Avenue, Cambridge CB2 0QH, England; c Institute for Cell and Molecular Biosciences, Newcastle University, Framlington Place, Newcastle upon Tyne NE2 1HH, England; d Science Technology and Facilities Council, Rutherford Appleton Laboratory, Didcot OX11 0FA, England; e Research Complex at Harwell, Rutherford Appleton Laboratory, Didcot OX11 0FA, England

**Keywords:** macromolecular crystallography, experimental phasing, machine learning, structure determination, phasing, X-ray crystallography

## Abstract

This study describes a method to estimate the likelihood of success in determining a macromolecular structure by X-ray crystallography and experimental single-wavelength anomalous dispersion (SAD) or multiple-wavelength anomalous dispersion (MAD) phasing based on initial data-processing statistics and sample crystal properties. Such a predictive tool can rapidly assess the usefulness of data and guide the collection of an optimal data set. The increase in data rates from modern macromolecular crystallography beamlines, together with a demand from users for real-time feedback, has led to pressure on computational resources and a need for smarter data handling. Statistical and machine-learning methods have been applied to construct a classifier that displays 95% accuracy for training and testing data sets compiled from 440 solved structures. Applying this classifier to new data achieved 79% accuracy. These scores already provide clear guidance as to the effective use of computing resources and offer a starting point for a personalized data-collection assistant.

## Abstract 2

Combining data-analysis statistics from crystallographic software with machine learning can predict the chances of experimental phasing success.

## 1. Introduction   

### 1.1. Protein crystallography   

For more than half a century, X-ray diffraction has been used to investigate protein crystals and the resulting diffraction images have been analysed to reveal the underlying structure of the protein to atomic detail. Despite well established techniques and dedicated user facilities, the vast majority of recorded diffraction data do not yield a protein structure (http://biosync.sbkb.org). For example, in 2016 it is estimated that less than 7% of diffraction data measured at European synchrotrons resulted in structures deposited in the Protein Data Bank (PDB; Berman *et al.*, 2000 ▸). This calculation is based on an average collection time of 5 min per data set and assuming 200 operational days a year with 23 h of runtime per day. The possible factors affecting whether data lead to a structure deposition or not are manifold: (i) the crystal material comprising the purified protein and the additional chemicals used to crystallize it; (ii) the beamline hardware and capabilities, which define the experiments that can be carried out; (iii) the data-collection strategy, which is determined based on (i) and (ii); and (iv) intensity integration and assessment of the quality of the measured data as well as phase estimation, the latter finally determining whether a data set results in a structure or not. Each of these factors can be represented by one or more metrics, in particular those describing the protein and those derived from data analysis. Use of these metrics offers a unique opportunity to predict the usefulness of a given data set, *i.e.* whether or not it will result in an atomic structure.

In this publication, we use machine learning and commonly applied statistical methods to analyse quality metrics from data analysis combined with protein sequence information. This serves as a basis for developing an interactive user guide to help crystallographers assess their data sets in order to determine which should be put forward for full analysis and structure solution using experimental phasing for phase estimation (Drenth, 1999 ▸; Dauter *et al.*, 2002 ▸; Blow & Rossmann, 1961 ▸; Blundell & Johnson, 1976 ▸). It is hoped that such a tool will enable structural biologists to better plan experiments and improve upon the estimated 7% success rate.

### 1.2. Machine learning   

Machine learning is part of the field of artificial intelligence. It uses statistical methods to develop algorithms which allow a computer to ‘learn’ in a data-driven manner and make predictions based on the learned information (Kohavi & Provost, 1998 ▸). ‘Learning’ implies that a task or prediction has not been hard-coded by a programmer in advance (Bishop, 2006 ▸). The main purpose of machine learning is to identify patterns in given training data and to predict an outcome for any new data based on the learned pattern.

The input data are usually held in a database, here METRIX_DB, and can be extracted in a tabular fashion, with columns and their headers giving the characteristics/features/dimensions of the data and each row representing a sample. Commonly, the data are split randomly into training and test sets, with the former being used to train a machine-learning algorithm and the latter being used to assess the performance of the finalized, trained model. Generally, *k*-fold cross-validation against the training set is performed to highlight any overfitting, which is monitored through classification accuracy. In supervised learning, the data have been annotated with labels of the known result, here representing two classes in a classification problem, and an equal distribution of class sizes is desirable. A confusion matrix is used for performance assessment, giving details about correctly identified positive (true positive; TP) and negative (true negative; TN) samples as well as wrong classifications (false positive, FP; false negative, FN). These classification outcomes are the basis on which to calculate additional metrics (classification accuracy and error, sensitivity, specificity, false-positive rate, precision, *F*_1_ score). Additionally, the area under a curve of a receiver operating characteristic (ROC) curve is calculated. A classification error of 5% is often used as a benchmark, as this is the typically observed human classification performance (Dodge & Karam, 2017 ▸).

In a pre-assessment step the most important features in decision making are identified using statistical tools such as Pearson’s linear correlation coefficients and recursive feature elimination. The use of this subset of features for classifier training improves the stability and performance of the classifier and reduces computation time (Pyle, 1999 ▸; Pang-Ning *et al.*, 2006 ▸; Guyon & Elisseeff, 2003 ▸). Training a classifier to create a predictive model is then an iterative process of training, testing and assessment until the desired stability and performance are reached.

In this case study, we focused on supervised learning to solve a binary classification problem, namely the likelihood of experimental phasing success (class label ‘1’ or positive) or failure (class label ‘0’ or negative). The algorithms used to create trained models are decision trees, random forest classifiers and their derivatives (Breiman *et al.*, 1984 ▸), and support vector machines (SVMs; Cortes & Vapnik, 1995 ▸).

## 2. Methods   

### 2.1. METRIX_DB database   

For the project that is described here, a database called METRIX_DB was created using the SQLite3 programming language accessed through a standard library within Python. At the time of writing, the database held the details of 810 released PDB structures. The diffraction images for these structures have been curated to match the set that was used to determine the published three-dimensional coordinates. At the moment, these structures stem from two structural genomics projects: 303 from the Structural Genomics Consortium (SGC; https://www.thesgc.org) at Oxford University, England and 507 from the Joint Center for Structural Genomics (JCSG; http://www.jcsg.org) at Stanford Synchrotron Radiation Lightsource, USA. We acknowledge that by using structures from two major laboratories, their distribution may not be entirely representative of the PDB.

For 364 of these structures the data were collected as ‘native’ and for 446 the data collection produced an anomalous MAD or SAD experiment. The data were acquired at both synchrotron and in-house facilities and therefore also cover a range of detectors, *i.e.* photon-counting and CCD cameras, as well as X-ray sources. The resolution for the structures ranges from 1.05 to 3.8 Å; soluble and membrane proteins are covered as well as proteins in complexes with other proteins, peptides or nucleic acids. The anomalous scatterer used in experimental phasing was introduced by means of protein production in a selenium-enriched medium to create selenomethionine (SeMet) in nearly all cases.

The metadata for these 810 structures were retrieved from the published PDB files and stored in METRIX_DB. Additional information was created when carrying out data integration and reduction, experimental phasing and sequence analysis. Where multiple wavelengths were available for a structure, the data set for each wavelength is considered a separate sample. After all of the data had been collected in the different tables of the database, individual columns containing the information of interest were selected and combined into a new table and exported as a file of comma-separated values which could directly be used in machine learning.

The code for the database can be found at https://github.com/ccp4/metrix-database.

### 2.2. Data-reduction and phasing pipeline   

Many of the details about the various data sets to be used in machine learning are statistics created during data reduction and phasing. Rather than executing these computational steps in a serial manner, a processing framework has been created using the Python 2.7 programming language to streamline the process using a compute cluster. A series of scripts has been developed to run *xia*2 (Winter, 2010 ▸) using *DIALS* (Winter *et al.*, 2018 ▸) and *AIMLESS* (Evans, 2006 ▸) for diffraction-image integration and data reduction. The statistics recorded in METRIX_DB are averages over the entire resolution range for a data set of a given wavelength. Although it is recognized (Usón & Sheldrick, 2018 ▸) that experimental phasing success can be sensitive to the high-resolution cutoff used, we chose not to investigate the resolution-dependence of the quality metrics included here owing to the increase of complexity for this proof-of-principle study. Only samples for which data reduction was successful were taken forward into experimental phasing.

For experimental phasing the *SHELXC*/*D*/*E* pipeline (Sheldrick, 2010 ▸) was used. If one wavelength was identified during data reduction, even if the data were collected as ‘native’, then a SAD experiment was assumed and phasing was carried out accordingly. If more than one wavelength was identified, the data were phased as a MAD experiment. Only samples for which the phasing software exited without error were used for machine learning and assigned a label, either ‘1’ or ‘0’, respectively, depending on whether the protein backbone could be traced or not. ‘Native’ data were not automatically assigned with label ‘0’, as several examples originally phased through molecular replacement also exhibited an anomalous signal strong enough for experimental phasing owing to intrinsic metals, for example in the active site.

With the exception of a hold-out set used to calibrate the best classifier, the concession was made to not check data-reduction and phasing output in depth, or optimize input parameters for each structure, in order to be able to run computations on a computational cluster and hence in a time-efficient manner. A total of 703 samples were used for training and testing the classifiers, and a further 34 for calibration before predicting with new samples.

### 2.3. Protein   

The sequence of each protein published alongside the structure was retrieved from the PDB and used for various calculations. For each sequence the molecular weight and number of atoms was calculated and stored in METRIX_DB. Using the unit-cell dimensions, molecular weight and the *MATTHEWS_COEF* tool from the *CCP*4 suite (Winn *et al.*, 2011 ▸; Matthews, 1968 ▸), the most likely number of molecules in the asymmetric unit was determined as well as the unit-cell volume and the solvent content. The number of anomalous scatterers expected to be present in the structure was determined by counting the methionines in the sequence and was multiplied by the number of molecules most likely to be found in the asymmetric unit. Overall, this gave reasonably good estimates for most samples, but did fail in cases of proteins in complexes and a few cases in which the anomalous scatterer was not selenium.

### 2.4. New test data   

The data used in this challenge were provided by the protein crystallography group at the University of Newcastle, England. None of the proteins analysed were present in the training or testing data. For 12 samples, the data collections were carried out on beamlines I03, I04, I04-1 and I24 at Diamond Light Source using a PILATUS detector. Data measured with this type of detector were available in the training and test sets. A further 12 samples were from a recent data collection on I04 using its new hardware setup of an EIGER detector and a multi-axis goniometer, for which no data were available in the training and test sets. The new diffraction data were integrated in the same way as the training and test data.

### 2.5. Machine learning   

The machine-learning aspect of this publication is based on Python 3.6. Other packages used are *pandas* 0.23.0 (McKinney, 2010 ▸), *Matplotlib* 2.2.3 (Hunter, 2007 ▸), *SciPy* 1.1.0 (Oliphant, 2007 ▸), *mlxtend* 0.13.0 (Raschka, 2018 ▸), *scikit-learn* 0.20.0 (Pedregosa *et al.*, 2011 ▸) and *NumPy* 1.14.3 (Oliphant, 2006 ▸).

The code for the machine-learning component of this publication can be found at https://github.com/ccp4/metrix_ml.

To ensure that the performance of a classifier is not biased to one particular class, it is important to have a balanced data set in which both classes are present equally. This also needs to be considered when splitting the data into training and testing sets, as the dominant class is likely to be more frequently found and will therefore skew the performance of any classifier to only be able to predict this class. However, in our case a balance between the classes was not achievable. Therefore, the split into training and testing data was stratified to ensure that the two sets are representative of the data, meaning that they maintain the class distribution. Here, we used a common split of 20% of data being assigned to the testing set and 80% remaining in a training set, while at the same time maintaining a class distribution of 66% for class ‘1’ and 33% for class ‘0’. A random seed has also been defined to ensure reproducibility when splitting the data in subsequent executions. Additionally, some classifiers allow weights to be assigned to the different classes to achieve a balance, which will be explored when carrying out a randomized search for the best hyperparameters (see below). This also applies to the hold-out set used to calibrate the classifier before prediction.

Overfitting means that the algorithm learns the training data and predicts these cases very well, apparently producing very good performance measures. However, challenged with a new, unseen sample the algorithm performs badly and fails to generalize. *k*-fold cross-validation for the training set was used to address this problem. Crucially, if the class distribution is unbalanced then this needs to be reflected in the cross-validation folds as well. In this study, we used a threefold cross-validation. The testing set is only used for assessing the trained, hyperparameter-optimized model at the very end.

For support vector machines, the data were standardized using the StandardScaler class from the pre-processing module of *scikit-learn* to scale to unit variance. For decision trees and random forest algorithms, however, standardization is not necessary.

A full list of all 44 features investigated here is given in Supplementary Table S1. This also includes the custom column transformations discussed in Appendix *A* [App appa]. All features investigated here were plotted against each other and their linear Pearson’s correlation coefficients, *r*, were determined using the corr() function in *pandas*. Any correlation coefficient which has an associated *p*-value of <0.05 can be considered to be meaningful and a correlation can be identified. The results were visualized in a correlation matrix with negative correlations (−1 to 0) coloured red and positive correlations (0 to 1) coloured blue. To quantify the correlation strength between two variables a coefficient of determination, *r* ^2^, was calculated. This gives the variance in the data of one dependent variable explained by the independent variable in the pair. An *r* ^2^ of >10% therefore indicates a weak correlation and an *r* ^2^ of >90% indicates a strong correlation. Only correlations that fulfil *p* < 0.05 and *r* ^2^ > 10% will be considered here.

For support vector machines feature importances are not readily available. Recursive feature elimination was therefore used to identify the most likely number of features as well as those most important for decision making. This was monitored through changes in classification accuracy upon the withdrawal of a feature. The recursive feature-elimination function with cross-validation from the feature_selection module in *scikit-learn* was used for this assessment.

The classifiers listed below have been used. For all classifiers the hyperparameters used have been determined in a randomized search using the RandomizedSearchCV function in *scikit-learn*, which tried 500 combinations for a given range. A basic scheme of training and assessment can be found in Supplementary Fig. S1. The following classifiers have been investigated for their suitability as a predictive tool for the available data: a support vector machine with a linear kernel and a radial-base function kernel, a decision tree, a decision tree with bagging, a decision tree with AdaBoost, a random forest and an extreme randomized forest. More details of the hyperparameter settings for the individual classifiers can be found in Appendix *B* [App appb]. The hyperparameters used for the best classifier after identifying the most important features are given in Supplementary Table S2. The metrics used to assess the different classifiers are detailed in Appendix *C* [App appc].

As mentioned previously, the data used in this study are unbalanced regarding class distributions. Although this distribution was maintained by using a stratified split when separating the test and training sets, this imbalance still had an effect on how reliable the predicted class probabilities were. In order to address this problem, the best classifier was calibrated with a hold-out set that was neither part of the test nor the training set using the CalibrateClassifierCV function in *scikit-learn* with cv=‘prefit’.

After calibrating the best classifier, the predict() function from the *scikit-learn* package was used to challenge it with new samples.

### 2.6. Comparison with an existing SAD prediction tool   

The software tool *plan_SAD_experiment* (Terwilliger *et al.*, 2016*a* ▸,*b* ▸) from the *Phenix* analysis package (version 1.17.1; Liebschner *et al.*, 2019 ▸) was used to calculate the probability of success for a given wavelength, assuming that each wavelength represents a SAD data set. The tool was executed with the following parameters: phenix.plan_sad_experiment seq_file=<PDB.fasta> atom_type=Se wavelength=<wave> resolution=<Dmin> data=<mtz_file>, where wavelength and resolution have been queried from METRIX_DB, and mtz_file contains the integrated intensities for each wavelength of a given PDB entry.

## 3. Results   

### 3.1. METRIX_DB database   

Based on the results of automated analysis, 440 structures successfully produced 703 samples, each of which is a crystallographic data set at a single wavelength. The class labels for these samples were verified through manual inspection of the automatic processing results, with 232 identified as class ‘0’ and 471 as class ‘1’. Information is held in METRIX_DB as a collection of tables, with each table relating to a stage of crystallographic data analysis, for example sequence details, data reduction, experimental phasing and the deposited PDB file information for reference. METRIX_DB is structured such that the number of samples and features to be investigated can easily be expanded. Measures have been put in place to expand the database in the future, with the aim to ultimately use the results from the Synchweb/ISPyB user interface (Fisher *et al.*, 2015 ▸), which is used to manage data-collection results during a visit.

### 3.2. Pre-assessment of the data   

**Figure 1:**
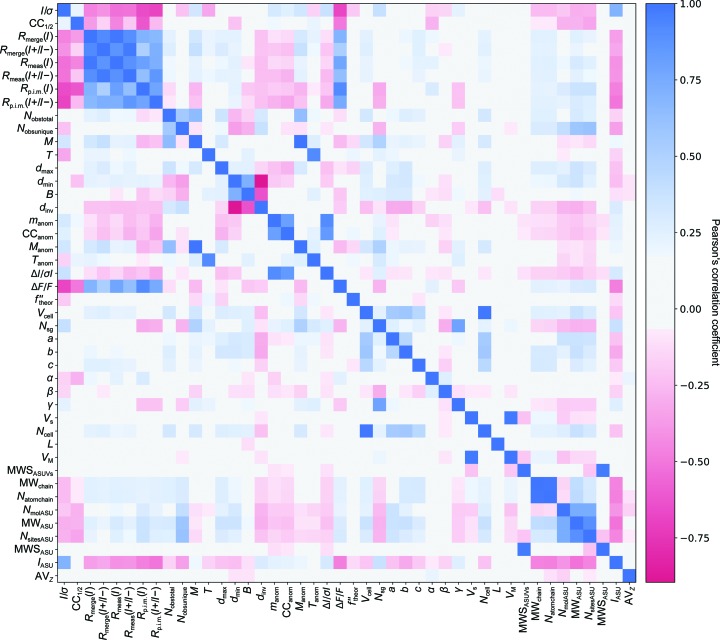
Correlation matrix of Pearson’s correlation coefficients between feature pairs to identify linear correlations between them. All 44 features investigated have been plotted. Blue indicates positive linear correlation ranging from 0 to 1 and red indicates negative linear correlation ranging from −1 to 0. The intensity of the colour indicates the strength of the correlation. All numerical values can be found in Supplementary Table S3.

Fig. 1 ▸ shows a correlation matrix for the linear Pearson’s correlation coefficients determined for all features. The features are grouped such that correlations between descriptors of diffraction data quality are concentrated in the top left quadrant and correlations between protein descriptors are located in the bottom right quadrant. The corresponding correlation coefficients (*r*), associated *p*-values and coefficients of determination (*r^2^*) as quantitative measures of the correlation strength are reported in Supplementary Table S3.

The highest scoring features for three decision trees, two random forests and the linear SVM classifiers are reported in Supplementary Table S4, with the corresponding performance statistics on the test set given in Supplementary Table S5.

Features related to and extracted from experimental phasing software, such as CC_weak_, CC_all_ and CFOM, were used in initial, exploratory work. These features were so dominant that the information provided from data integration and scaling statistics would vanish. However, the purpose of this study was to identify indicators for experimental phasing success at the step of data integration and scaling, so experimental phasing statistics were excluded from further analysis.

**Figure 2:**
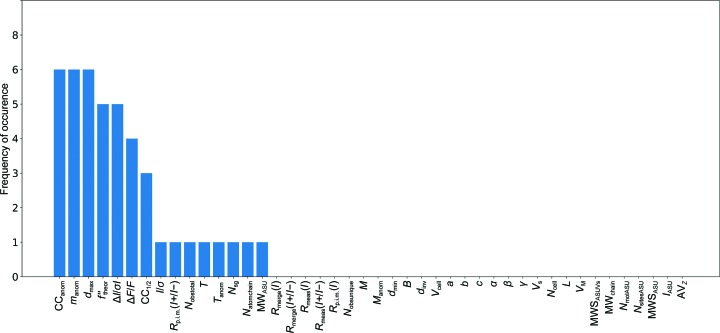
Bar plot of feature occurrences found during the initial classifier training. Features that are important in the decision-making process during classification appear more frequently regardless of which classifier has been used. The highest scoring features for the individual classifiers can be found in Supplementary Table S4. The most frequently found features are CC_anom_, Δ*I*/σ*I*, *m*_anom_, *d*_max_, Δ*F*/*F*, *f*′′_theor_ and CC_1/2_.

The most important features in the decision-making process in the different classifiers and the frequency of appearance of a particular feature were counted and plotted in Fig. 2 ▸. For retraining the classifiers, the six highest scoring features were chosen: CC_anom_, Δ*I*/σ*I*, *m*_anom_, *d*_max_, Δ*F*/*F* and *f*′′_theor_. Smaller and larger feature sets based on the scores plotted in Fig. 2 ▸, using one feature (CC_anom_), two features (CC_anom_, *m*_anom_; CC_anom_, *I*/σ), five features (CC_anom_, *m*_anom_, *d*_max_, Δ*F*/*F*, *f*′′_theor_) and seven features (CC_anom_, Δ*I*/σ*I*, *m*_anom_, *d*_max_, Δ*F*/*F*, *f*′′_theor_, CC_1/2_), were also tried (data not shown), but none of the resulting classifiers performed as well as the decision tree with AdaBoost and the six highest scoring features.

### 3.3. Feature correlations   

A closer look at the correlation matrix identifies high degrees of positive or negative correlation between the data-quality descriptors commonly used by crystallographers. These statistics either assess the precision of unmerged (*R*_merge_, *R*_meas_) or merged (*I*/σ, CC_1/2_, *R*_p.i.m._) intensities. The pattern of correlations for *R*_merge_ and *R*_meas_ are very similar, supporting the view that the introduction of *R*_meas_ renders *R*_merge_ obsolete (Weiss & Hilgenfeld, 1997 ▸). For the merged intensity precision indicators, there are broadly similar patterns (with the sign of the correlation coefficient inverted for *R*_p.i.m._ compared with *I*/σ or CC_1/2_), but the differences between these patterns suggest that distinct information is expressed by these metrics. The relations between these quantities have been discussed elsewhere (Karplus & Diederichs, 2015 ▸). It should be mentioned that the spread of multiplicity, *M*, in METRIX_DB is limited and therefore a relationship with *I*/σ could not be explored without artificial truncation of data sets, which we did not perform. Indicators of anomalous signal in the data (Δ*I*/σ*I*, Δ*F*/*F*, CC_anom_ and *m*_anom_) are only weakly correlated to the theoretical anomalous scattering factor *f*′′_theor_, presumably because of other factors influencing the observed signal such as anomalous scatterer occupancy and *B* factor, and the overall signal-to-noise ratio in the data. The anomalous signal as expressed by Δ*F*/*F* is clearly reflected in strong correlations to *R*_meas_(*I*), *R*_merge_(*I*) and *R*_p.i.m._(*I*) calculated assuming intensity equivalence of Bijvoet mates, *i.e.* higher values when compared with *R*_merge_(*I*+/*I*−), *R*_meas_(*I*+/*I*−) and *R*_p.i.m._(*I*+/*I*−) that account for the presence of anomalous signal.

Other commonly used metrics, such as *N*_obstotal_, *N*_obsunique_, *d*_min_ and *B*, show lower level correlations to data-quality descriptors. *d*_max_, however, is typically defined by a backstop shadow rather than the intrinsic quality of the measured intensities and is uncorrelated to data-quality descriptors. Data completeness, *T*, is given here by a single value, which ignores the potentially relevant effect of systematic patterns of incompleteness, such as missing wedges, shadowed regions of the detector and a variable high-resolution cutoff at different regions of the detector. More detailed analysis such as this will require more sophisticated descriptors of completeness and a more representative database.

There are weak correlations between descriptors of the protein content and data-quality indicators, for example MWS_ASU_, which gives the ratio between molecular weight and the number of anomalous scatterers in the asymmetric unit, or *I*_ASU_, which represents the ratio between signal (*I*/σ) and asymmetric unit content (MW_ASU_). As METRIX_DB expands it will be interesting to explore these relationships, but at this stage we avoid speculative interpretation. The pattern of correlations within the group of protein-content descriptors shows some larger features, as expected for metrics that are all used to quantify various aspects of the crystal content.

Unit-cell parameters are weakly correlated with various parameters but display no strong predictive properties. Also visible, as one would expect, are correlations between space-group number (*N*_sg_), multiplicity (*M*) and *I*/σ through its relation to multiplicity.

### 3.4. Selecting the best-performing classifier   

**Figure 3:**
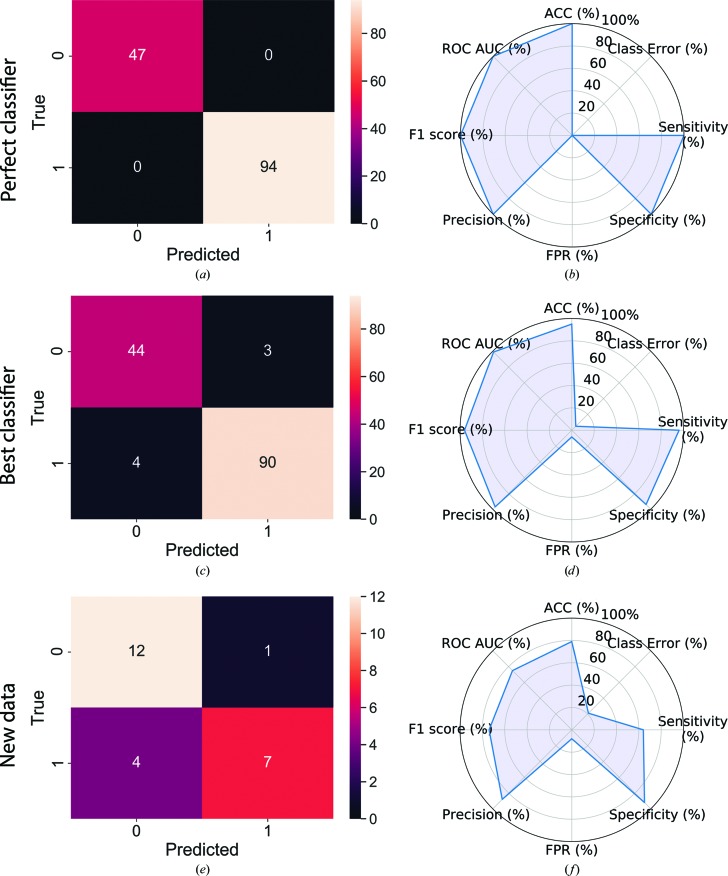
Confusion matrices and radar plots for a perfect classifier (*a*, *b*), the best classifier, a decision tree with AdaBoost (*c*, *d*), and for new data (*e*, *f*) and the performance of the best classifiers on new data. The confusion matrices (*a*, *c*, *e*) give the scores for the four possible classification outcomes: true negative at the top left, true positive at the bottom right, false negative at the top right and false positive at the bottom left. The perfect classifier has no misclassifications, whereas the decision tree with AdaBoost places three class ‘0’ samples and four class ‘1’ samples into the wrong category. For the new data one sample has been identified as false positive and four as false negatives. The classification outcomes serve as a basis to calculate classification accuracy (ACC), classification error (Class Error), sensitivity (Sensitivity), specificity (Specificity), false-positive rate (FPR), precision (Precision) and *F*_1_ score (F1 score) as they are plotted in the radar plots (*b*, *d*, *f*). The value ROC AUC is determined by calculating the area under the curve of an ROC curve.

The reduced feature set identified above was used to retrain all classifiers, and their performance results on the test set are given in Supplementary Table S6. The best-performing classifier was a decision tree with AdaBoost, and its confusion matrix and radar plot are shown in Figs. 3 ▸(*c*) and 3 ▸(*d*), respectively. Additionally, the results for a perfect classifier are given for comparison [Figs. 3 ▸(*a*) and 3 ▸(*b*)].

This classifier is the best-performing classifier based on the assessment metrics used, achieving a classification accuracy of 95%. The sensitivity, or true-positive rate, was found to be 96% (90 out of 94 samples) and the specificity, or true-negative rate, was 94% (44 out of 47 samples). The false-positive rate was 6% (three samples) with precision 97%. The *F*_1_ score was 96% and the area under the curve of an ROC curve was 99%.

### 3.5. Testing the prediction classifier against new data   

The performance metric results for the new data using the decision tree with AdaBoost are given in Supplementary Table S7 and the corresponding confusion matrix and radar plot in Figs. 3 ▸(*e*) and 3 ▸(*f*). A total of 24 new samples were used to challenge the classifier. The samples comprised proteins not present in METRIX_DB, and the data-collection strategies and beamline hardware were entirely different to those used for the training data. These therefore presented a significant challenge for the classifier. The experimental outcomes for these new samples were assessed and labelled by the user and were only revealed after prediction had been carried out. A probability threshold of 80.0% for class ‘1’ was applied, reflecting the fact that users would typically prefer a low false-positive rate, *i.e.* have some confidence that class ‘1’ truly reflects a successful structure determination.

The classification accuracy achieved was 79%. Sensitivity and specificity were 64% (seven out of 11 samples) and 92% (12 out of 13 samples), respectively. The false-positive rate was 8% (one out of 13 samples) with a precision of 86% and an *F*_1_ score of 74%, and the area under the curve of a receiver operating characteristic curve (ROC AUC) was determined as 75%.

### 3.6. Comparison with an existing SAD prediction tool   

The same samples, a total of 703, that were used as training and testing sets for machine learning were analysed by *phenix.plan_SAD_experiment*. A probability threshold of 80% for SAD phasing success was chosen as a measure of confidence in the prediction, as was performed for the new user sample (see Section 3.5). Overall, the tool achieved a classification accuracy of 68%. The vast majority of true-positive samples were correctly identified by the prediction tool, with a sensitivity of 97%. Many of the false-negative samples had a wavelength chosen for low-energy remote data collection as part of a MAD data set where there is weak or no anomalous signal but that was essential to solve the phase problem. Of the true-negative samples, 21 were correctly identified, which is reflected in a false-positive rate of 91%. In comparison, the false-positive rate for our testing set is 5% and 8% for new user data. We stress that the results from the two approaches are not directly comparable as the tools are intended for different purposes. *Phenix.plan_SAD_ experiment* was designed to advise a user who has already chosen to try SAD phasing whether they are going to be successful, which it does very well based on the sensitivity of 97%. However, its purpose is not to identify data sets for which data were collected either as native or MAD, hence the false-positive rate of 91%.

## 4. Discussion   

Analysing crystallographic results with the aim to predict the likely experimental phasing success using machine learning is a data-driven approach. As such, the outcome is defined by the kind of data that have been used in training. In this study, we have chosen to focus on particular experimental phasing approaches represented by a training database of native, SAD and MAD data sets. The content of METRIX_DB is currently limited to published structures where data are publicly available. Nearly all of the crystallographic data used here exhibited anomalous signal that made experimental phasing straightforward. A post-mortem analysis of a collection of weak S-SAD data sets is under way with the aim of including such data in METRIX_DB. Ultimately, representative data from each kind of data collection performed by users needs to be included in METRIX_DB. This should reduce the constraints currently imposed on the content of METRIX_DB and therefore on the scope of our studies. Additionally, this would close the technology gap between the data currently measured on modern X-ray beamlines and those contained in METRIX_DB. For example, current synchrotron data sets are almost exclusively measured using photon-counting hybrid pixel detectors and fine-slicing methods. However, our analysis clearly provides initial insight into the potential application of machine learning in protein crystallography to assist a scientist during decision making in experimental phasing. For future investigations METRIX_DB will be expanded to make use of other descriptors, for example, results from analysis and prediction tools making use of the protein sequence. Furthermore, recent changes in data policy for many European synchrotrons will allow user data to be incorporated into training databases, making them more relevant and effective.

Clearly, the highest scoring features identified here, Δ*I*/σ*I*, Δ*F*/*F*, CC_anom_, *m*_anom_, *d*_max_ and *f*′′_theor_, should be optimized by a crystallographer prior to, or during, data collection and analysis, whether or not a classifier is being used to provide guidance. For example, to maximize *f*′′_theor_ a wavelength scan should be carried out prior to data collection to select an optimal wavelength. To optimize *d*_max_ an additional low-resolution pass could be collected and/or the beamstop size and position could be set to ensure low-resolution data coverage. Regarding Δ*I*/σ*I*, Δ*F*/*F*, CC_anom_ and *m*_anom_ it would be advisable to look at the classifier prediction for experimental phasing success and continue to collect additional rotation images in order to increase anomalous signal while monitoring radiation damage. Alternatively, data collected from several crystals of the same protein with the same anomalous scatterer can be combined.

The matrix of Pearson’s linear correlation coefficients showed that a subset of data-quality metrics [*R*_merge_, *R*_meas_, *R*_p.i.m._, *R*_merge_(*I*+/*I*−), *R*_meas_(*I*+/*I*−) and *R*_p.i.m._(*I*+/*I*−)] are highly correlated with each other and hence convey very similar information. This gives additional support to previously published analysis (Karplus & Diederichs, 2012 ▸; Diederichs & Karplus, 2013 ▸; Evans & Murshudov, 2013 ▸) describing the relationships between these metrics.

Conducting an in-depth analysis of the resolution dependence of many of the metrics investigated here, in particular those identified as being most important when judging the likelihood of experimental phasing success using a machine-learning tool, was not within the scope of this manuscript but will be part of further work. In our study, all of the statistics were averages across the entire resolution range of a given sample, where the high-resolution limit was set by the data-integration software. Generally, phasing techniques do not use the full resolution range of data but typically truncate the data to a lower resolution limit for substructure determination. Using a systematic approach by applying common cutoffs to all data sets would therefore be useful in identifying the resolution range that gives the highest chance of success, regardless of the actual resolution limit of the data.

The best classifier as judged by its performance metrics presented here is a decision tree with AdaBoost. With a classification error of 5%, this classifier performs at about the same level as a human would when presented with the test data (Dodge & Karam, 2017 ▸). The small number of false positives and false negatives, four and three, respectively, should allow the classifier to generalize when challenged with a novel sample. This is further detailed below.

Many of the data sets present in the training and test sets were measured with CCD and photon-counting hybrid pixel-array detectors (PADs) using typical crystal rotation ranges per image of >0.5° (wide-slicing). The 24 data sets used to test the classifier were, however, measured at Diamond Light Source on PADs using fine-slicing (typically <0.1°). This difference in data-collection approach may be one reason for the lower classification accuracy of 79%. In general, however, one would always expect a reduction in accuracy for any classifier when used with new data. Surprisingly, perhaps, the classifier performs well in correctly identifying samples where experimental phasing is likely to fail, class ‘0’, with a specificity of 92%. A broader representation of detector types and data-collection strategies in METRIX_DB would be likely to result in better classifier performance against new data and could highlight other high-scoring features to optimize.

Similarly, the use of different anomalous scatterers in the diffraction experiment needs to be considered since all derivatized training samples here were selenomethionine proteins. Samples 9 to 12 of the new user data were heavy-atom soaks using platinum, gold or lead compounds. Samples 9 and 10 were correctly classified as class ‘0’, since a lack of anomalous signal meant experimental phasing failed. This was probably owing to poor incorporation of the heavy atoms during soaking. Of the remaining two samples, one was classified correctly as class ‘1’ (sample 11).

Although attempted, a direct comparison with the already available *phenix.plan_SAD_experiment* is not justified as this tool was specifically designed to help crystallographers maximize their chances of solving the phase problem with a SAD experiment. However, our machine-learning approach is designed to work in a more general way by looking at the data measured for different phasing methods.

**Figure 4:**
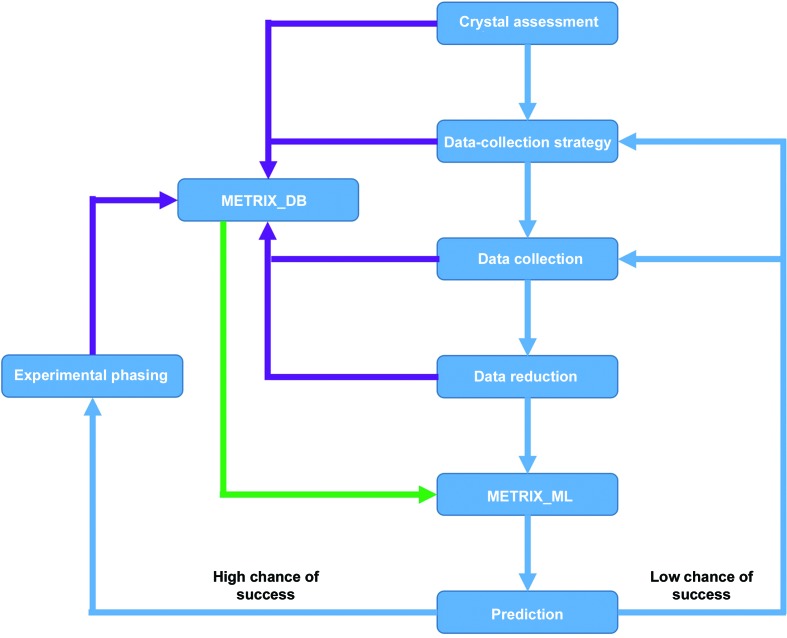
General workflow envisaged for an interactive user assistant. Blue depicts the different steps in structure solution from diffraction data collection to experimental phasing. Dark purple gives the feedback and statistics of every step, which is stored in the database METRIX_DB. Green represents the statistics stored in METRIX_DB which are used to train the classifier in METRIX_ML.

An implementation of an interactive user guide can be envisaged in which the classifier is trained with standard data sets, makes predictions on incoming data collections and reports results to the user. Feedback can be given directly through the Synchweb/ISPyB interface as part of the general data-analysis workflow. After assessing their results, either while still at the beamline or later after more careful analysis, users annotate the data through Synchweb/ISPyB with the actual experimental outcome by simply clicking on a box to set a label. This data would then be included in METRIX_DB or extracted directly from Synchweb/ISPyB to retrain the classifier. The retraining process itself would be carried out during shutdown periods when no new user data are acquired or between visits, depending on computational resources. Over time, such a classifier would be customized towards the proteins investigated by a certain user group and their typical data-collection experimental phasing strategies. A classifier would become more stable and the training frequency can then be reduced. The flowchart in Fig. 4 ▸ gives a schematic outline for an interactive user system. Although a user will always be able to ignore the recommendations and trigger data analysis manually, including our trained algorithm in the analysis pipelines is expected to help in balancing the workload on the computing infrastructure in a more intelligent way than the brute-force approach currently in use.

Additionally, we envision a system in which a classifier executes repeat predictions on incomplete data while data collection is still ongoing to indicate a trend of success and to identify the point at which the data are sufficient to attempt experimental phasing. This would be very beneficial, for example, in the case where multiple partial rotation data sets are being collected and combined. Post-mortem analysis regarding such an application is under way using S-SAD data.

We have presented a proof of principle for how machine learning can be used in protein crystallography, in particular for experimental phasing, and have discussed the possible applications of such predictive classifiers. This concept will be generalized in the future to cover a broader range of structure-determination methods including isomorphous replacement-related methods and molecular replacement. This will require a substantial expansion of METRIX_DB.

Although intervention by an expert crystallographer is still essential for corner cases, such machine-learning support systems will become more and more important. The data rates and data volumes accumulated during diffraction experiments are already such that it is difficult for a human to keep pace. Furthermore, the number of scientists who are using protein crystallo­graphy as an analytical tool rather than a scientific discipline is rapidly increasing, placing a greater burden on automated acquisition and analysis systems at user facilities. For these reasons, it is expected that decision-making tools based on machine learning will form an integral part of macromolecular crystallography beamline facilities in the future.

## 5. Related literature   

The following references are cited in the supporting information for this article: Arndt *et al.* (1968 ▸), Bijvoet *et al.* (1951 ▸), Diederichs & Karplus (1997 ▸), Howell & Smith (1992 ▸), Schneider & Sheldrick (2002 ▸), Srinivasan & Parthasarthy (1976 ▸), Weiss (2001 ▸) and Wilson (1942 ▸, 1949 ▸, 1950 ▸).

## Supplementary Material

Supplementary tables and figure. DOI: 10.1107/S2052252520000895/jt5042sup1.pdf

## Appendix A Custom column transformations

The custom column transformations are largely based on knowledge of the protein and are inspired by the publication by Holton & Frankel (2010 ▸).

The molecular weight in the asymmetric unit is defined as

where MW_chain_ is the molecular weight of the protein (Da) and *N*_molASU_ is the number of molecules in the asymmetric unit as determined by the Matthews coefficient.

The expected number of anomalous scatterers is estimated as

where *N*_methionine_ is the number of methionines identified from the sequence file and *N*_molASU_ is the expected number of molecules in the asymmetric unit as determined by the Matthews coefficient.

The ratio between the molecular weight and the number of anomalous scatterer sites in the asymmetric unit is given by

where MW_ASU_ is defined as in (1) and *N*_sitesASU_ is defined as in (2).

MW_ASU_ has also been looked at in the context of solvent content,

where MWS_ASU_ is given by (2) and the solvent content *V*_s_ has been determined through the Matthews coefficient.

The molecular weight of the protein has been brought into context with the number of atoms in its chain,

where MW_chain_ is the molecular weight (Da) of the protein and *N*_atomchain_ is the number of atoms in the protein based on the sequence including hydrogens.

Based on *N*_cell_ (Holton & Frankel, 2010 ▸), we calculate the total number of ordered atoms in the unit cell (including hydrogens) as follows,

where *V*_cell_ is the unit-cell volume, *V*_s_ is given by the Matthews coefficient and AV_*Z*_ is as defined in (5).

A ratio between the measured signal *I*/σ and the molecular weight in the asymmetric unit was created through

The Wilson *B* factor, *B*, as extracted from the data-processing statistics, was multiplied by a constant (−2) to give −*B* = −2*B*, and *d*_inv_ is defined as

*−B*, *d*_inv_ and *N*_cell_ are then combined as follows,

## Appendix B Classifier hyperparameter details

### b1. Support vector machine with linear kernel

The SVC() function from the svm class in *scikit-learn* was used with a linear kernel. ‘C’ and a ‘class_weight’ of either ‘none’ or ‘balanced’ were the only hyperparameters to be optimized for this classifier.

### b.2. Support vector machine with radial basis function (RBF) kernel

This allows the classification of samples where a simple linear separation cannot be achieved. The SVC() function from the svm class in *scikit-learn* was used with an RBF kernel. Hyperparameters ‘C’, ‘gamma’ and a ‘class_weight’ of either ‘none’ or ‘balanced’ were optimized.

### b3. Decision tree

For each decision at the *i*th node in a decision tree all features available are evaluated and the one that leads to the cleanest split, separating most of the samples as judged by either Gini purity *G_i_* or entropy reduction *H_i_*, is applied. A node is considered to be ‘pure’ according to the Gini criterion with gini = 0 if all of the samples in it belong to the same class or if the entropy is reaching zero if the highest level of order is achieved.

where *p*_*i*,*k*_ is the ratio between the instances of the different classes *k* out of all samples *n*.

The underlying algorithm is for classification and regression trees (CART; Breiman *et al.*, 1984 ▸) and the following hyperparameters have been optimized in a randomized search.

‘criterion’: splitting criterion (Gini purity and entropy reduction).
‘max_features’: the maximum number of features to use throughout the tree or base estimator when carrying out a split [all features (44) in the first round and between two and seven, whichever is closest to the square root of the number of features].
‘min_samples_split’: the minimum number of samples in a node needed to consider a split (between two and 20).
‘max_depth’: the maximum number of splits that can be in a tree, also known as the depth of the tree (between five and 10).
‘min_samples_leaf’: the minimum number of samples needed to create a leaf (between one and 20).
‘max_leaf_nodes’: the maximum number of leaves in the tree; the tree stops splitting when the maximum number is reached (between 10 and 20).

Additionally, ‘class_weight’ was set to ‘balanced’.

### b4. Decision tree with bagging

Also known as bootstrap aggregating, this is a higher level application to improve the stability of a decision-tree classifier, reduce its variance and avoid overfitting (Breiman, 1996 ▸). The samples have been drawn with replacement. The base estimator in the application used here is a decision tree for which the abovementioned hyperparameters have been optimized by a randomized search, but additionally the number of classifiers (‘n_estimators’, between 100 and 10 000) was also evaluated and ‘class_weight’ could be either ‘balanced’ or ‘none’.

### b5. Decision tree with AdaBoost

AdaBoost, like bagging, is an ensemble method but uses sequential learning. The base estimator used here is a decision tree for which the hyperparameters listed above have been optimized in a randomized search and additionally the number of estimators used (‘n_estimators’, between 100 and 10 000) has been searched for as well. Furthermore, the ‘class_weight’ could be either ‘balanced’ or ‘none’ and a ‘learning_rate’ was also set. The particular AdaBoost algorithm used here is ‘Stagewise Additive Modelling using a Multiclass Exponential loss function’, SAMME.R (where R stands for ‘Real’), which uses class probabilities rather than predictions as would be used with SAMME (Zhu *et al.*, 2009 ▸).

### b6. Random forest

Random forest is an ensemble method using decision trees as base estimators and trained using a bagging method (Ho, 1995 ▸). The samples used to build a tree are only a subset and are drawn with replacement. All the hyperparameters given for the decision-tree classifier above have been used and additionally ‘class_weights’ could be either ‘balanced’ or ‘none’ and ‘n_estimator’ could be between 100 and 10 000.

### b7. Extreme random forest

Additionally to using a subset of features, the threshold for a feature at which this split occurs is chosen randomly rather than looking for the best threshold (Geurts *et al.*, 2006 ▸). In the application here, the decision-tree hyperparameters given above were optimized, except for ‘max_depth’, which was set to 1. Additionally, the ‘class_weights’ could be either ‘balanced’ or ‘none’ and ‘n_estimators’ could be between 100 and 10 000.

## Appendix C Classifier assessment

To assess any machine-learning result a confusion matrix of the classification outcomes was created (true positive, TP; true negative, TN; false positive, FP; false negative, FN), which was used to calculate a series of performance indicators. All positive samples (P = TP + FN) are those labelled as class ‘1’ and all negative samples (N = TN + FP) are those labelled as class ‘0’.

‘Classification accuracy’ (ACC) is defined as

and shows how well the model does in predicting the correct class for both positive (class ‘1’) and negative (class ‘0’) cases.

The ‘classification error’, on the other hand, gives details about how many samples have been attributed to the wrong class and is defined as

‘Sensitivity’, recall or true-positive rate (TPR) defines how well the model does in identifying samples of the positive class correctly out of all positive samples and is determined as

‘Specificity’ or true-negative rate (TNR), on the other hand, gives the tendency of the model to correctly identify those samples that belong to class ‘0’ out of all negative samples and is defined as

False-positive rate (FPR) gives the number of true negatives or samples of class ‘0’ that have been predicted as positives out of all negative samples:

‘Precision’ or positive predictive value (PPV) is defined as the number of true positive samples to be predicted as positive out of all samples being classified as belonging to class ‘1’:

‘F1 score’ is a trade-off between ‘precision’ and ‘sensitivity’ and is defined as

In a good classifier this should be close to 100%, but can be significantly lower depending on whether a classifier is desired to have high precision or sensitivity. For an ideal model, the ‘classification error’ and ‘FPR’ would both be zero or 0%, and the ‘sensitivity’, ‘specificity’ and ‘precision’ would be one or 100%. In general, a model cannot achieve perfect predictions unless it has learned the data. A classification error of ∼5% is usually found for a human, and a good classifier should be close or better than this value (Dodge & Karam, 2017 ▸).

A visual way to judge the performance of a model is using a receiver operating characteristic (ROC) curve. In such a plot the false-positive rate, FPR, is plotted on the horizontal axes whereas the true-positive rate, TPR, is plotted on the vertical axes. A well performing model will exhibit a curve that peaks near the top left corner where FPR is minimal and TPR is at its maximum. Additionally, the area under the curve (AUC) of an ROC curve can be determined, with a well performing model having an AUC value close to 1 or 100% (Hanley & McNeil, 1983 ▸).

## References

[bb1] Arndt, U. W., Crowther, R. A. & Mallett, J. F. W. (1968). *J. Phys. E Sci. Instrum.* **1**, 510–516. 10.1088/0022-3735/1/5/303 5317723

[bb2] Berman, H. M., Westbrook, J., Feng, Z., Gilliland, G., Bhat, T. N., Weissig, H., Shindyalov, I. N. & Bourne, P. E. (2000). *Nucleic Acids Res.* **28**, 235–242. 10.1093/nar/28.1.235 PMC102472 10592235

[bb3] Bijvoet, J. M., Peerdeman, A. F. & van Bommel, A. J. (1951). *Nature*, **168**, 271–272.

[bb4] Bishop, C. M. (2006). *Pattern Recognition and Machine Learning.* New York: Springer.

[bb5] Blow, D. M. & Rossmann, M. G. (1961). *Acta Cryst.* **14**, 1195–1202.

[bb6] Blundell, T. L. & Johnson, L. N. (1976). *Protein Crystallography.* New York: Academic Press.

[bb7] Breiman, L. (1996). *Mach. Learn.* **24**, 123–140.

[bb8] Breiman, L., Friedman, J. H., Olshen, R. A. & Stone, C. J. (1984). *Classification and Regression Trees.* London: Taylor & Francis.

[bb9] Cortes, C. & Vapnik, V. N. (1995). *Mach. Learn.* **20**, 273–797.

[bb10] Dauter, Z., Dauter, M. & Dodson, E. J. (2002). *Acta Cryst.* D**58**, 494–506. 10.1107/s090744490200118x 11856836

[bb11] Diederichs, K. & Karplus, P. A. (1997). *Nat. Struct. Biol.* **4**, 269–275. 10.1038/nsb0497-269 9095194

[bb12] Diederichs, K. & Karplus, P. A. (2013). *Acta Cryst.* D**69**, 1215–1222. 10.1107/S0907444913001121 PMC3689524 23793147

[bb13] Dodge, S. & Karam, L. (2017). *arXiv*:1705.02498.

[bb14] Drenth, J. (1999). *Principles of Protein X-ray Crystallography.* New York: Springer.

[bb15] Evans, P. (2006). *Acta Cryst.* D**62**, 72–82. 10.1107/S0907444905036693 16369096

[bb16] Evans, P. R. & Murshudov, G. N. (2013). *Acta Cryst.* D**69**, 1204–1214. 10.1107/S0907444913000061 PMC3689523 23793146

[bb17] Fisher, S. J., Levik, K. E., Williams, M. A., Ashton, A. W. & McAuley, K. E. (2015). *J. Appl. Cryst.* **48**, 927–932. 10.1107/S1600576715004847 PMC4453979 26089766

[bb18] Geurts, P., Ernst, D. & Wehenkel, L. (2006). *Mach. Learn.* **63**, 3–42.

[bb19] Guyon, I. & Elisseeff, A. (2003). *J. Mach. Learn. Res.* **3**, 1157–1182.

[bb20] Hanley, J. A. & McNeil, B. J. (1983). *Radiology*, **148**, 839–843. 10.1148/radiology.148.3.6878708 6878708

[bb21] Ho, T. K. (1995). *Proceedings of 3rd International Conference on Document Analysis and Recognition*, Vol. 1, pp. 278–282. Piscataway: IEEE.

[bb22] Holton, J. M. & Frankel, K. A. (2010). *Acta Cryst.* D**66**, 393–408. 10.1107/S0907444910007262 PMC2852304 20382993

[bb23] Howell, P. L. & Smith, G. D. (1992). *J. Appl. Cryst.* **25**, 81–86.

[bb24] Hunter, J. D. (2007). *Comput. Sci. Eng.* **9**, 90–95.

[bb25] Karplus, P. A. & Diederichs, K. (2012). *Science*, **336**, 1030–1033. 10.1126/science.1218231 PMC3457925 22628654

[bb26] Karplus, P. A. & Diederichs, K. (2015). *Curr. Opin. Struct. Biol.* **34**, 60–68. 10.1016/j.sbi.2015.07.003 PMC4684713 26209821

[bb27] Kohavi, R. & Provost, F. (1998). *Mach. Learn.* **30**, 271–274.

[bb51] Liebschner, D., Afonine, P. V., Baker, M. L., Bunkóczi, G., Chen, V. B., Croll, T. I., Hintze, B., Hung, L.-W., Jain, S., McCoy, A. J., Moriarty, N. W., Oeffner, R. D., Poon, B. K., Prisant, M. G., Read, R. J., Richardson, J. S., Richardson, D. C., Sammito, M. D., Sobolev, O. V., Stockwell, D. H., Terwilliger, T. C., Urzhumtsev, A. G., Videau, L. L., Williams, C. J. & Adams, P. D. (2019). *Acta Cryst.* D**75**, 861–877. 10.1107/S2059798319011471 PMC6778852 31588918

[bb28] Matthews, B. W. (1968). *J. Mol. Biol.* **33**, 491–497. 10.1016/0022-2836(68)90205-2 5700707

[bb29] McKinney, W. (2010). *Proceedings of the 9th Python in Science Conference*, edited by S. van der Walt & J. Millman, pp. 51–56. Austin: SciPy Society.

[bb30] Oliphant, T. E. (2006). *Guide to NumPy.* Spanish Fork: Trelgol Publishing.

[bb31] Oliphant, T. E. (2007). *Comput. Sci. Eng.* **9**, 10–20.

[bb32] Pang-Ning, T., Steinbach, M. & Kumar, V. (2006). *Introduction to Data Mining.* Harlow: Pearson Education.

[bb33] Pedregosa, F., Varoquaux, G., Gramfort, A., Michel, V., Thirion, B., Grisel, O., Blondel, M., Prettenhofer, P., Weiss, R., Dubourg, V., Vanderplas, J., Passos, A., Cournapeau, D., Brucher, M., Perrot, M. & Duchesnay, É. (2011). *J. Mach. Learn. Res.* **12**, 2825–2830.

[bb34] Pyle, D. (1999). *Data Preparation for Data Mining.* San Francisco: Morgan Kaufmann.

[bb35] Raschka, S. (2018). *J. Open Source. Softw.* **3**, 638–639.

[bb36] Schneider, T. R. & Sheldrick, G. M. (2002). *Acta Cryst.* D**58**, 1772–1779. 10.1107/s0907444902011678 12351820

[bb37] Sheldrick, G. M. (2010). *Acta Cryst.* D**66**, 479–485. 10.1107/S0907444909038360 PMC2852312 20383001

[bb38] Srinivasan, R. & Parthasarthy, S. (1976). *Some Statistical Applications in X-ray Crystallography.* Oxford: Pergamon Press.

[bb39] Terwilliger, T. C., Bunkóczi, G., Hung, L.-W., Zwart, P. H., Smith, J. L., Akey, D. L. & Adams, P. D. (2016*a*). *Acta Cryst.* D**72**, 346–358. 10.1107/S2059798315019269 PMC4784666 26960122

[bb40] Terwilliger, T. C., Bunkóczi, G., Hung, L.-W., Zwart, P. H., Smith, J. L., Akey, D. L. & Adams, P. D. (2016*b*). *Acta Cryst.* D**72**, 359–374. 10.1107/S2059798315019403 PMC4784667 26960123

[bb41] Usón, I. & Sheldrick, G. M. (2018). *Acta Cryst.* D**74**, 106–116. 10.1107/S2059798317015121 PMC5947774 29533236

[bb42] Weiss, M. S. (2001). *J. Appl. Cryst.* **34**, 130–135.

[bb43] Weiss, M. S. & Hilgenfeld, R. (1997). *J. Appl. Cryst.* **30**, 203–205.

[bb44] Wilson, A. J. C. (1942). *Nature*, **150**, 152.

[bb45] Wilson, A. J. C. (1949). *Acta Cryst.* **2**, 318–321.

[bb46] Wilson, A. J. C. (1950). *Acta Cryst.* **3**, 397–398.

[bb47] Winn, M. D., Ballard, C. C., Cowtan, K. D., Dodson, E. J., Emsley, P., Evans, P. R., Keegan, R. M., Krissinel, E. B., Leslie, A. G. W., McCoy, A., McNicholas, S. J., Murshudov, G. N., Pannu, N. S., Potterton, E. A., Powell, H. R., Read, R. J., Vagin, A. & Wilson, K. S. (2011). *Acta Cryst.* D**67**, 235–242. 10.1107/S0907444910045749 PMC3069738 21460441

[bb48] Winter, G. (2010). *J. Appl. Cryst.* **43**, 186–190.

[bb49] Winter, G., Waterman, D. G., Parkhurst, J. M., Brewster, A. S., Gildea, R. J., Gerstel, M., Fuentes-Montero, L., Vollmar, M., Michels-Clark, T., Young, I. D., Sauter, N. K. & Evans, G. (2018). *Acta Cryst.* D**74**, 85–97. 10.1107/S2059798317017235 PMC5947772 29533234

[bb50] Zhu, J., Zou, H., Rosset, S. & Hastie, T. (2009). *Stat. Interface*, **2**, 349–360.

