# Supplementary material for: The predictive power of data-processing statistics
Source: IUCrJ. 2020 Feb 27;7(Pt 2):342–54. doi: 10.1107/S2052252520000895 (PMC7055369; doi:10.1107/S2052252520000895)

IUCrJ

Volume 7 (2020)

Supporting information for article:

Melanie Vollmar, James M. Parkhurst, Dominic Jaques, Arnaud Baslé, Garib N. Murshudov, David G. Waterman and Gwyndaf Evans

Table S1 Complete feature list for all features investigated in this publication.

Name	Explanation
I/σ	$<I/\sigma>_{obs}$, Signal (intensity I) to noise (uncertainty or measurement error σ) ratio (Wilson, 1949; Srinivasan & Parthasarthy, 1976)[1]
$CC_{1/2}$	$CC_{1/2}$ (Karplus & Diederichs, 2012; Diederichs & Karplus, 2013; Evans & Murshudov, 2013)
$R_{merge}I$	R_{sym} or R_{merge} (Arndt *et al.*, 1968; Wilson, 1950). Determined by treating Friedel pair I+ and I- as equivalent measurements.
$R_{merge}(I+/I-)$	R_{merge} determined by treating Friedel pair I+ and I- as independent measurements.
$R_{meas}I$	R_{meas} or $R_{r.i.m.}$ (Diederichs & Karplus, 1997; Weiss, 2001). Determined by treating Friedel pair I+ and I- as equivalent measurements.
$R_{meas}(I+/I-)$	R_{meas} determined by treating Friedel pair I+ and I- as independent measurements.
$R_{p.i.m.}I$	$R_{p.i.m.}$ (Weiss, 2001). Determined by treating Friedel pair I+ and I- as equivalent measurements.
$R_{p.i.m.}(I+/I-)$	$R_{p.i.m.}$ determined by treating Friedel pair I+ and I- as independent measurements.
$N_{obstotal}$	Total number of observations
$N_{obsunique}$	Total number of unique observations
M	Multiplicity; Number of observations for each reflection
T	Completeness; Representation of missing data
d_{max}	Low resolution limit found by integration software
d_{min}	High resolution limit found by integration software
B	Wilson B-factor (Wilson, 1942)
m_{anom}	Anomalous slope (Howell & Smith, 1992)
CC_{anom}	CC_{anom} (Schneider & Sheldrick, 2002)
M_{anom}	Anomalous multiplicity; Number of observations for each anomalous reflection
T_{anom}	Anomalous Completeness; Representation of missing anomalous data
$\Delta I/\sigma I$	Signal to noise ratio for the anomalous differences

ΔF/F	structure factors for the anomalous differences (Bijvoet *et al.*, 1951)
f''_{theor}	Calculated with CROSSEC (Winn *et al.*, 2011)
V_{cell}	Unit cell volume
N_{sg}	Space group number from data reduction
a	Unit cell parameter a
b	Unit cell parameter b
c	Unit cell parameter c
α	Unit cell parameter alpha
β	Unit cell parameter beta
γ	Unit cell parameter gamma
V_m	Matthews coefficient
V_S	Expected solvent content determined using Matthews coefficient
N_{cell}	Defined in (6)
N_{molASU}	Expected number of molecules in the asymmetric unit determined using Matthews coefficient
MW_{ASU}	Defined in (1)
$N_{sitesASU}$	Defined in (2)
MW_{chain}	Molecular weight of the protein based on its sequence
$N_{atomchain}$	Number of atoms in the protein based on its sequence
MWS_{ASU}	Defined in (3)
MWS_{ASUVs}	Defined in (4)
AV_z	Defined in (5)
I_{ASU}	Defined in (7)
d_{inv}	Defined in (8)
L	Defined in (9)

[1] where I is the observed intensity and σ its associated uncertainty provided $\chi^2 \sim 1$ for observed and estimated differences between symmetry-related observations

Table S2 Hyperparameter settings for the best classifier, a decision tree with AdaBoost, based on performance statistics.

Decision tree with AdaBoost	Base_estimator:<br>class_weight = balanced<br>criterion = entropy<br>max_depth = 3<br>max_features = 2<br>max_leaf_nodes = 17<br>min_samples_leaf = 8<br>min_samples_split = 18<br>random_state = 0<br><br>AdaBoostClassifier:<br>n_estimators = 5694<br>learning_rate = 0.6355<br>algorithm = SAMME.R<br>random_state = 5

Table S3 Numerical results for Pearson's correlation coefficient.

Feature	Feature	r	p	r2
I/σ	$CC_{1/2}$	0.204099	0.000001	0.041657
I/σ	$R_{merge}I$	-0.461005	0	0.212525
I/σ	R_{merge}(I+/I-)	-0.406516	0	0.165255
I/σ	$R_{meas}I$	-0.524406	0	0.275002
I/σ	R_{meas}(I+/I-)	-0.51533	0	0.265565
I/σ	$R_{p.i.m.}I$	-0.658338	0	0.433409
I/σ	$R_{p.i.m.}$(I+/I-)	-0.684956	0	0.469164
I/σ	$N_{obstotal}$	0.05358	0.204695	0.002871
I/σ	$N_{obsunique}$	-0.215612	0	0.046489
I/σ	M	0.327343	0	0.107153
I/σ	T	-0.319765	0	0.10225
I/σ	d_{max}	-0.004266	0.919627	0.000018

I/σ	d_{min}	-0.03209	0.447704	0.00103
I/σ	B	0.088631	0.035677	0.007855
I/σ	d_{inv}	0.082296	0.051186	0.006773
I/σ	m_{anom}	0.317502	0	0.100807
I/σ	CC_{anom}	0.254705	0	0.064875
I/σ	M_{anom}	0.340871	0	0.116193
I/σ	T_{anom}	-0.161448	0.000121	0.026065
I/σ	ΔI/σI	0.372491	0	0.13875
I/σ	ΔF/F	-0.673614	0	0.453755
I/σ	f'_{theor}	-0.1989	0.000002	0.039561
I/σ	V_{cell}	-0.005937	0.888309	0.000035
I/σ	N_{sg}	0.389966	0	0.152073
I/σ	a	-0.021461	0.611666	0.000461
I/σ	b	-0.043566	0.30254	0.001898
I/σ	c	-0.006508	0.877662	0.000042
I/σ	α	-0.164493	0.000089	0.027058
I/σ	β	-0.151351	0.000317	0.022907
I/σ	γ	0.228871	0	0.052382
I/σ	V_s	-0.04504	0.286468	0.002029
I/σ	N_{cell}	-0.004917	0.907409	0.000024
I/σ	L	0.028798	0.495661	0.000829
I/σ	V_m	-0.016452	0.697139	0.000271
I/σ	MWS_{ASUVs}	0.072105	0.087679	0.005199
I/σ	MW_{chain}	-0.246298	0	0.060663
I/σ	$N_{atomchain}$	-0.245889	0	0.060461
I/σ	N_{molASU}	-0.204453	0.000001	0.041801
I/σ	MW_{ASU}	-0.290423	0	0.084346
I/σ	$N_{sitesASU}$	-0.281477	0	0.07923
I/σ	MWS_{ASU}	0.044644	0.290734	0.001993
I/σ	I_{ASU}	0.699194	0	0.488872

I/σ	AV_z	0.022952	0.587147	0.000527
$CC_{1/2}$	$R_{merge}I$	-0.364135	0	0.132594
$CC_{1/2}$	$R_{merge}(I+/I-)$	-0.164214	0.000092	0.026966
$CC_{1/2}$	$R_{meas}I$	-0.451295	0	0.203667
$CC_{1/2}$	$R_{meas}(I+/I-)$	-0.204826	0.000001	0.041954
$CC_{1/2}$	$R_{p.i.m.}I$	-0.621068	0	0.385726
$CC_{1/2}$	$R_{p.i.m.}(I+/I-)$	-0.256568	0	0.065827
$CC_{1/2}$	$N_{obstotal}$	0.069437	0.100089	0.004821
$CC_{1/2}$	$N_{obsunique}$	-0.02572	0.542869	0.000662
$CC_{1/2}$	M	0.075758	0.072728	0.005739
$CC_{1/2}$	T	0.138029	0.001036	0.019052
$CC_{1/2}$	d_{max}	-0.037902	0.369796	0.001437
$CC_{1/2}$	d_{min}	-0.216243	0	0.046761
$CC_{1/2}$	B	0.09398	0.025886	0.008832
$CC_{1/2}$	d_{inv}	0.140168	0.000862	0.019647
$CC_{1/2}$	m_{anom}	0.171091	0.000046	0.029272
$CC_{1/2}$	CC_{anom}	0.133635	0.001497	0.017858
$CC_{1/2}$	M_{anom}	0.070818	0.0935	0.005015
$CC_{1/2}$	T_{anom}	0.204663	0.000001	0.041887
$CC_{1/2}$	ΔI/σI	0.091764	0.029618	0.008421
$CC_{1/2}$	ΔF/F	-0.498313	0	0.248316
$CC_{1/2}$	f'_{theor}	-0.023131	0.584235	0.000535
$CC_{1/2}$	V_{cell}	-0.014342	0.734414	0.000206
$CC_{1/2}$	N_{sg}	0.104343	0.013329	0.010888
$CC_{1/2}$	a	-0.011953	0.777372	0.000143
$CC_{1/2}$	b	-0.041557	0.325411	0.001727
$CC_{1/2}$	c	-0.046993	0.266058	0.002208
$CC_{1/2}$	α	-0.275457	0	0.075877
$CC_{1/2}$	β	-0.038344	0.36424	0.00147

$CC_{1/2}$	γ	0.059401	0.159637	0.003528
$CC_{1/2}$	V_s	0.00842	0.842123	0.000071
$CC_{1/2}$	N_{cell}	-0.013873	0.742795	0.000192
$CC_{1/2}$	L	-0.002407	0.954594	0.000006
$CC_{1/2}$	V_m	0.016777	0.691467	0.000281
$CC_{1/2}$	MWS_{ASUVs}	0.036719	0.384942	0.001348
$CC_{1/2}$	MW_{chain}	-0.068916	0.102669	0.004749
$CC_{1/2}$	$N_{atomchain}$	-0.068311	0.105729	0.004666
$CC_{1/2}$	N_{molASU}	-0.286824	0	0.082268
$CC_{1/2}$	MW_{ASU}	-0.277251	0	0.076868
$CC_{1/2}$	$N_{sitesASU}$	-0.325971	0	0.106257
$CC_{1/2}$	MWS_{ASU}	0.035462	0.401431	0.001258
$CC_{1/2}$	I_{ASU}	0.130181	0.001985	0.016947
$CC_{1/2}$	AV_z	0.002417	0.954413	0.000006
$R_{merge}I$	$R_{merge}(I+/I-)$	0.915797	0	0.838685
$R_{merge}I$	$R_{meas}I$	0.986226	0	0.972642
$R_{merge}I$	$R_{meas}(I+/I-)$	0.896073	0	0.802947
$R_{merge}I$	$R_{p.i.m.}I$	0.697979	0	0.487175
$R_{merge}I$	$R_{p.i.m.}(I+/I-)$	0.66775	0	0.445891
$R_{merge}I$	$N_{obstotal}$	0.231511	0	0.053597
$R_{merge}I$	$N_{obsunique}$	0.009718	0.818191	0.000094
$R_{merge}I$	M	0.321401	0	0.103299
$R_{merge}I$	T	0.203856	0.000001	0.041557
$R_{merge}I$	d_{max}	0.233883	0	0.054701
$R_{merge}I$	d_{min}	0.22442	0	0.050364
$R_{merge}I$	B	-0.042683	0.312457	0.001822
$R_{merge}I$	d_{inv}	-0.225517	0	0.050858
$R_{merge}I$	m_{anom}	-0.1013	0.016292	0.010262
$R_{merge}I$	CC_{anom}	-0.126364	0.002691	0.015968

$R_{merge}I$	M_{anom}	0.317057	0	0.100525
$R_{merge}I$	T_{anom}	0.21886	0	0.0479
$R_{merge}I$	$\Delta I/\sigma I$	-0.117	0.005486	0.013689
$R_{merge}I$	$\Delta F/F$	0.692511	0	0.479571
$R_{merge}I$	f'_{theor}	0.042779	0.311371	0.00183
$R_{merge}I$	V_{cell}	0.243134	0	0.059114
$R_{merge}I$	N_{sg}	0.06547	0.121074	0.004286
$R_{merge}I$	a	0.133692	0.00149	0.017874
$R_{merge}I$	b	0.195158	0.000003	0.038087
$R_{merge}I$	c	0.255679	0	0.065372
$R_{merge}I$	α	0.191732	0.000005	0.036761
$R_{merge}I$	β	-0.017561	0.67783	0.000308
$R_{merge}I$	γ	0.033298	0.430792	0.001109
$R_{merge}I$	V_s	0.045625	0.280249	0.002082
$R_{merge}I$	N_{cell}	0.22952	0	0.052679
$R_{merge}I$	L	-0.031771	0.452241	0.001009
$R_{merge}I$	V_m	0.045035	0.286519	0.002028
$R_{merge}I$	MWS_{ASUVs}	-0.010808	0.798219	0.000117
$R_{merge}I$	MW_{chain}	0.245374	0	0.060208
$R_{merge}I$	$N_{atomchain}$	0.248079	0	0.061543
$R_{merge}I$	N_{molASU}	0.144846	0.000572	0.02098
$R_{merge}I$	MW_{ASU}	0.258505	0	0.066825
$R_{merge}I$	$N_{sitesASU}$	0.209098	0.000001	0.043722
$R_{merge}I$	MWS_{ASU}	0.005793	0.891002	0.000034
$R_{merge}I$	I_{ASU}	-0.360897	0	0.130247
$R_{merge}I$	AV_z	-0.048875	0.247363	0.002389
$R_{merge}(I+/I-)$	$R_{meas}I$	0.872964	0	0.762067
$R_{merge}(I+/I-)$	$R_{meas}(I+/I-)$	0.977293	0	0.955101
$R_{merge}(I+/I-)$	$R_{p.i.m.}I$	0.508323	0	0.258392

R_{merge}(I+/I-)	$R_{p.i.m.}$(I+/I-)	0.723511	0	0.523468
R_{merge}(I+/I-)	$N_{obstotal}$	0.271626	0	0.073781
R_{merge}(I+/I-)	$N_{obsunique}$	0.028581	0.498925	0.000817
R_{merge}(I+/I-)	M	0.386217	0	0.149163
R_{merge}(I+/I-)	T	0.203553	0.000001	0.041434
R_{merge}(I+/I-)	d_{max}	0.287031	0	0.082387
R_{merge}(I+/I-)	d_{min}	0.211205	0	0.044608
R_{merge}(I+/I-)	B	0.016579	0.694915	0.000275
R_{merge}(I+/I-)	d_{inv}	-0.221175	0	0.048918
R_{merge}(I+/I-)	m_{anom}	-0.200151	0.000002	0.04006
R_{merge}(I+/I-)	CC_{anom}	-0.232355	0	0.053989
R_{merge}(I+/I-)	M_{anom}	0.383715	0	0.147237
R_{merge}(I+/I-)	T_{anom}	0.184825	0.00001	0.03416
R_{merge}(I+/I-)	ΔI/σI	-0.211527	0	0.044744
R_{merge}(I+/I-)	ΔF/F	0.517815	0	0.268132
R_{merge}(I+/I-)	f'_{theor}	0.001603	0.969757	0.000003
R_{merge}(I+/I-)	V_{cell}	0.262157	0	0.068726
R_{merge}(I+/I-)	N_{sg}	0.107384	0.010853	0.011531
R_{merge}(I+/I-)	a	0.152656	0.000281	0.023304
R_{merge}(I+/I-)	b	0.170265	0.00005	0.02899
R_{merge}(I+/I-)	c	0.255633	0	0.065348
R_{merge}(I+/I-)	α	0.068077	0.106929	0.004634
R_{merge}(I+/I-)	β	-0.070144	0.09667	0.00492
R_{merge}(I+/I-)	γ	0.040317	0.34006	0.001625
R_{merge}(I+/I-)	V_s	0.071711	0.089429	0.005142
R_{merge}(I+/I-)	N_{cell}	0.246402	0	0.060714
R_{merge}(I+/I-)	L	-0.026914	0.524305	0.000724
R_{merge}(I+/I-)	V_m	0.072527	0.085833	0.00526
R_{merge}(I+/I-)	MWS_{ASUVs}	0.017661	0.6761	0.000312
R_{merge}(I+/I-)	MW_{chain}	0.227518	0	0.051764

R_{merge}(I+/I-)	$N_{atomchain}$	0.232133	0	0.053886
R_{merge}(I+/I-)	N_{molASU}	0.10473	0.012988	0.010968
R_{merge}(I+/I-)	MW_{ASU}	0.203019	0.000001	0.041217
R_{merge}(I+/I-)	$N_{sitesASU}$	0.133579	0.001504	0.017843
R_{merge}(I+/I-)	MWS_{ASU}	0.038638	0.360577	0.001493
R_{merge}(I+/I-)	I_{ASU}	-0.323916	0	0.104922
R_{merge}(I+/I-)	AV_z	-0.061284	0.14679	0.003756

R_{meas}I	R_{meas}(I+/I-)	0.88109	0	0.77632
R_{meas}I	$R_{p.i.m.}$I	0.805114	0	0.648208
R_{meas}I	$R_{p.i.m.}$(I+/I-)	0.72241	0	0.521876
R_{meas}I	$N_{obstotal}$	0.170748	0.000047	0.029155
R_{meas}I	$N_{obsunique}$	0.025188	0.551248	0.000634
R_{meas}I	M	0.219009	0	0.047965
R_{meas}I	T	0.167228	0.000068	0.027965
R_{meas}I	d_{max}	0.192321	0.000004	0.036987
R_{meas}I	d_{min}	0.238619	0	0.056939
R_{meas}I	B	-0.083135	0.048855	0.006911
R_{meas}I	d_{inv}	-0.237285	0	0.056304
R_{meas}I	m_{anom}	-0.128236	0.00232	0.016444
R_{meas}I	CC_{anom}	-0.15076	0.000335	0.022729
R_{meas}I	M_{anom}	0.214824	0	0.046149
R_{meas}I	T_{anom}	0.156749	0.000191	0.02457
R_{meas}I	ΔI/σI	-0.144988	0.000565	0.021021
R_{meas}I	ΔF/F	0.777715	0	0.60484
R_{meas}I	f'_{theor}	0.056476	0.181247	0.003189
R_{meas}I	V_{cell}	0.208189	0.000001	0.043343
R_{meas}I	N_{sg}	-0.014872	0.724994	0.000221
R_{meas}I	a	0.109518	0.009368	0.011994
R_{meas}I	b	0.171806	0.000042	0.029517

$R_{meas}I$	c	0.224827	0	0.050547
$R_{meas}I$	α	0.227705	0	0.05185
$R_{meas}I$	β	0.00499	0.906035	0.000025
$R_{meas}I$	γ	-0.023322	0.581135	0.000544
$R_{meas}I$	V_s	0.030983	0.463528	0.00096
$R_{meas}I$	N_{cell}	0.196883	0.000003	0.038763
$R_{meas}I$	L	-0.029689	0.482426	0.000881
$R_{meas}I$	V_m	0.026196	0.535429	0.000686
$R_{meas}I$	MWS_{ASUVs}	-0.014152	0.7378	0.0002
$R_{meas}I$	MW_{chain}	0.251862	0	0.063435
$R_{meas}I$	$N_{atomchain}$	0.253493	0	0.064259
$R_{meas}I$	N_{molASU}	0.187036	0.000008	0.034983
$R_{meas}I$	MW_{ASU}	0.296993	0	0.088205
$R_{meas}I$	$N_{sitesASU}$	0.258252	0	0.066694
$R_{meas}I$	MWS_{ASU}	0.001005	0.981026	0.000001
$R_{meas}I$	I_{ASU}	-0.401506	0	0.161207
$R_{meas}I$	AV_z	-0.044427	0.293085	0.001974
$R_{meas}(I+/I-)$	$R_{p.i.m.}I$	0.622879	0	0.387978
$R_{meas}(I+/I-)$	$R_{p.i.m.}(I+/I-)$	0.852228	0	0.726293
$R_{meas}(I+/I-)$	$N_{obstotal}$	0.188124	0.000007	0.035391
$R_{meas}(I+/I-)$	$N_{obsunique}$	0.059154	0.161384	0.003499
$R_{meas}(I+/I-)$	M	0.23533	0	0.05538
$R_{meas}(I+/I-)$	T	0.186294	0.000009	0.034706
$R_{meas}(I+/I-)$	d_{max}	0.22784	0	0.051911
$R_{meas}(I+/I-)$	d_{min}	0.222298	0	0.049416
$R_{meas}(I+/I-)$	B	-0.02576	0.542244	0.000664
$R_{meas}(I+/I-)$	d_{inv}	-0.239272	0	0.057251
$R_{meas}(I+/I-)$	m_{anom}	-0.23946	0	0.057341
$R_{meas}(I+/I-)$	CC_{anom}	-0.277271	0	0.076879

R_{meas}(I+/I-)	M_{anom}	0.23217	0	0.053903
R_{meas}(I+/I-)	T_{anom}	0.132299	0.001671	0.017503
R_{meas}(I+/I-)	ΔI/σI	-0.258223	0	0.066679
R_{meas}(I+/I-)	ΔF/F	0.622482	0	0.387484
R_{meas}(I+/I-)	f'_{theor}	0.028456	0.500797	0.00081
R_{meas}(I+/I-)	V_{cell}	0.215539	0	0.046457
R_{meas}(I+/I-)	N_{sg}	-0.011153	0.791926	0.000124
R_{meas}(I+/I-)	a	0.130941	0.001867	0.017146
R_{meas}(I+/I-)	b	0.135218	0.001313	0.018284
R_{meas}(I+/I-)	c	0.212645	0	0.045218
R_{meas}(I+/I-)	α	0.077456	0.066522	0.005999
R_{meas}(I+/I-)	β	-0.026945	0.523824	0.000726
R_{meas}(I+/I-)	γ	-0.041936	0.321008	0.001759
R_{meas}(I+/I-)	V_s	0.052772	0.211621	0.002785
R_{meas}(I+/I-)	N_{cell}	0.202852	0.000001	0.041149
R_{meas}(I+/I-)	L	-0.023343	0.580798	0.000545
R_{meas}(I+/I-)	V_m	0.047229	0.263665	0.002231
R_{meas}(I+/I-)	MWS_{ASUVs}	0.019086	0.651631	0.000364
R_{meas}(I+/I-)	MW_{chain}	0.244632	0	0.059845
R_{meas}(I+/I-)	$N_{atomchain}$	0.247478	0	0.061245
R_{meas}(I+/I-)	N_{molASU}	0.148335	0.000418	0.022003
R_{meas}(I+/I-)	MW_{ASU}	0.243234	0	0.059163
R_{meas}(I+/I-)	$N_{sitesASU}$	0.181835	0.000014	0.033064
R_{meas}(I+/I-)	MWS_{ASU}	0.039004	0.356038	0.001521
R_{meas}(I+/I-)	I_{ASU}	-0.398936	0	0.15915
R_{meas}(I+/I-)	AV_z	-0.053703	0.203656	0.002884
$R_{p.i.m.}$I	$R_{p.i.m.}$(I+/I-)	0.77852	0	0.606093
$R_{p.i.m.}$I	$N_{obstotal}$	-0.125573	0.002863	0.015769
$R_{p.i.m.}$I	$N_{obsunique}$	0.079253	0.060437	0.006281

$R_{p.i.m.}I$	M	-0.264174	0	0.069788
$R_{p.i.m.}I$	T	0.023045	0.585627	0.000531
$R_{p.i.m.}I$	d_{max}	-0.016903	0.689267	0.000286
$R_{p.i.m.}I$	d_{min}	0.227469	0	0.051742
$R_{p.i.m.}I$	B	-0.205812	0.000001	0.042359
$R_{p.i.m.}I$	d_{inv}	-0.223638	0	0.050014
$R_{p.i.m.}I$	m_{anom}	-0.186272	0.000009	0.034697
$R_{p.i.m.}I$	CC_{anom}	-0.200395	0.000002	0.040158
$R_{p.i.m.}I$	M_{anom}	-0.267631	0	0.071626
$R_{p.i.m.}I$	T_{anom}	-0.070019	0.097265	0.004903
$R_{p.i.m.}I$	ΔI/σI	-0.210635	0	0.044367
$R_{p.i.m.}I$	ΔF/F	0.911637	0	0.831082
$R_{p.i.m.}I$	f'_{theor}	0.110413	0.008801	0.012191
$R_{p.i.m.}I$	V_{cell}	0.017425	0.680201	0.000304
$R_{p.i.m.}I$	N_{sg}	-0.317886	0	0.101052
$R_{p.i.m.}I$	a	-0.00415	0.921799	0.000017
$R_{p.i.m.}I$	b	0.042599	0.313416	0.001815
$R_{p.i.m.}I$	c	0.059814	0.156746	0.003578
$R_{p.i.m.}I$	α	0.290484	0	0.084381
$R_{p.i.m.}I$	β	0.101562	0.016016	0.010315
$R_{p.i.m.}I$	γ	-0.21991	0	0.048361
$R_{p.i.m.}I$	V_s	-0.03449	0.414462	0.00119
$R_{p.i.m.}I$	N_{cell}	0.019206	0.649593	0.000369
$R_{p.i.m.}I$	L	-0.015183	0.719479	0.000231
$R_{p.i.m.}I$	V_m	-0.053071	0.209036	0.002817
$R_{p.i.m.}I$	MWS_{ASUVs}	-0.0234	0.579873	0.000548
$R_{p.i.m.}I$	MW_{chain}	0.22464	0	0.050463
$R_{p.i.m.}I$	$N_{atomchain}$	0.221729	0	0.049164
$R_{p.i.m.}I$	N_{molASU}	0.288049	0	0.082972
$R_{p.i.m.}I$	MW_{ASU}	0.363838	0	0.132378

$R_{p.i.m.}I$	$N_{sitesASU}$	0.369649	0	0.13664
$R_{p.i.m.}I$	MWS_{ASU}	-0.017649	0.676312	0.000311
$R_{p.i.m.}I$	I_{ASU}	-0.472569	0	0.223322
$R_{p.i.m.}I$	AV_z	-0.017299	0.682389	0.000299
$R_{p.i.m.}(I+/I-)$	$N_{obstotal}$	-0.078875	0.061679	0.006221
$R_{p.i.m.}(I+/I-)$	$N_{obsunique}$	0.122534	0.003622	0.015015
$R_{p.i.m.}(I+/I-)$	M	-0.218326	0	0.047666
$R_{p.i.m.}(I+/I-)$	T	0.115008	0.006344	0.013227
$R_{p.i.m.}(I+/I-)$	d_{max}	0.03876	0.359055	0.001502
$R_{p.i.m.}(I+/I-)$	d_{min}	0.204123	0.000001	0.041666
$R_{p.i.m.}(I+/I-)$	B	-0.120539	0.004215	0.01453
$R_{p.i.m.}(I+/I-)$	d_{inv}	-0.234282	0	0.054888
$R_{p.i.m.}(I+/I-)$	m_{anom}	-0.283278	0	0.080247
$R_{p.i.m.}(I+/I-)$	CC_{anom}	-0.328675	0	0.108027
$R_{p.i.m.}(I+/I-)$	M_{anom}	-0.222573	0	0.049539
$R_{p.i.m.}(I+/I-)$	T_{anom}	-0.009289	0.826082	0.000086
$R_{p.i.m.}(I+/I-)$	ΔI/σI	-0.317837	0	0.10102
$R_{p.i.m.}(I+/I-)$	ΔF/F	0.754161	0	0.568759
$R_{p.i.m.}(I+/I-)$	f'_{theor}	0.097987	0.02016	0.009601
$R_{p.i.m.}(I+/I-)$	V_{cell}	0.048119	0.254767	0.002315
$R_{p.i.m.}(I+/I-)$	N_{sg}	-0.292046	0	0.085291
$R_{p.i.m.}(I+/I-)$	a	0.047292	0.263028	0.002237
$R_{p.i.m.}(I+/I-)$	b	0.021151	0.616832	0.000447
$R_{p.i.m.}(I+/I-)$	c	0.071138	0.092025	0.005061
$R_{p.i.m.}(I+/I-)$	α	0.086378	0.040658	0.007461
$R_{p.i.m.}(I+/I-)$	β	0.077106	0.067766	0.005945
$R_{p.i.m.}(I+/I-)$	γ	-0.219592	0	0.048221
$R_{p.i.m.}(I+/I-)$	V_s	-0.005094	0.904101	0.000026
$R_{p.i.m.}(I+/I-)$	N_{cell}	0.047421	0.261733	0.002249

$R_{p.i.m.}$(I+/I-)	L	-0.011156	0.79187	0.000124
$R_{p.i.m.}$(I+/I-)	V_m	-0.023568	0.577155	0.000555
$R_{p.i.m.}$(I+/I-)	MWS_{ASUVs}	0.017605	0.677071	0.00031
$R_{p.i.m.}$(I+/I-)	MW_{chain}	0.240245	0	0.057718
$R_{p.i.m.}$(I+/I-)	$N_{atomchain}$	0.238626	0	0.056942
$R_{p.i.m.}$(I+/I-)	N_{molASU}	0.222534	0	0.049521
$R_{p.i.m.}$(I+/I-)	MW_{ASU}	0.294963	0	0.087003
$R_{p.i.m.}$(I+/I-)	$N_{sitesASU}$	0.262585	0	0.068951
$R_{p.i.m.}$(I+/I-)	MWS_{ASU}	0.030437	0.47145	0.000926
$R_{p.i.m.}$(I+/I-)	I_{ASU}	-0.503697	0	0.25371
$R_{p.i.m.}$(I+/I-)	AV_z	-0.028296	0.503215	0.000801
$N_{obstotal}$	$N_{obsunique}$	0.551436	0	0.304081
$N_{obstotal}$	M	0.646415	0	0.417853
$N_{obstotal}$	T	0.135979	0.001232	0.01849
$N_{obstotal}$	d_{max}	0.353478	0	0.124946
$N_{obstotal}$	d_{min}	-0.264105	0	0.069751
$N_{obstotal}$	B	-0.157408	0.000179	0.024777
$N_{obstotal}$	d_{inv}	0.312384	0	0.097584
$N_{obstotal}$	m_{anom}	-0.007891	0.851925	0.000062
$N_{obstotal}$	CC_{anom}	-0.034142	0.419198	0.001166
$N_{obstotal}$	M_{anom}	0.631109	0	0.398299
$N_{obstotal}$	T_{anom}	0.182437	0.000013	0.033283
$N_{obstotal}$	ΔI/σI	-0.015412	0.715425	0.000238
$N_{obstotal}$	ΔF/F	-0.115216	0.00625	0.013275
$N_{obstotal}$	f'_{theor}	-0.038106	0.367234	0.001452
$N_{obstotal}$	V_{cell}	0.321535	0	0.103385
$N_{obstotal}$	N_{sg}	0.092174	0.028896	0.008496
$N_{obstotal}$	a	0.189737	0.000006	0.036
$N_{obstotal}$	b	0.267943	0	0.071793

$N_{obstotal}$	c	0.240543	0	0.057861
$N_{obstotal}$	α	0.057847	0.170859	0.003346
$N_{obstotal}$	β	-0.031299	0.45899	0.00098
$N_{obstotal}$	γ	0.007018	0.868158	0.000049
$N_{obstotal}$	V_s	0.032405	0.44326	0.00105
$N_{obstotal}$	N_{cell}	0.322035	0	0.103707
$N_{obstotal}$	L	-0.00295	0.944364	0.000009
$N_{obstotal}$	V_m	0.011746	0.781126	0.000138
$N_{obstotal}$	MWS_{ASUVs}	-0.028153	0.505373	0.000793
$N_{obstotal}$	MW_{chain}	0.257968	0	0.066548
$N_{obstotal}$	$N_{atomchain}$	0.258263	0	0.0667
$N_{obstotal}$	N_{molASU}	0.118339	0.004969	0.014004
$N_{obstotal}$	MW_{ASU}	0.302447	0	0.091474
$N_{obstotal}$	$N_{sitesASU}$	0.255817	0	0.065442
$N_{obstotal}$	MWS_{ASU}	-0.01409	0.738917	0.000199
$N_{obstotal}$	I_{ASU}	-0.168933	0.000057	0.028538
$N_{obstotal}$	AV_z	0.003146	0.940673	0.00001
$N_{obsunique}$	M	-0.088718	0.035496	0.007871
$N_{obsunique}$	T	0.023936	0.571226	0.000573
$N_{obsunique}$	d_{max}	0.180039	0.000018	0.032414
$N_{obsunique}$	d_{min}	-0.356636	0	0.12719
$N_{obsunique}$	B	-0.294421	0	0.086684
$N_{obsunique}$	d_{inv}	0.38273	0	0.146482
$N_{obsunique}$	m_{anom}	-0.05534	0.190196	0.003062
$N_{obsunique}$	CC_{anom}	-0.090669	0.031627	0.008221
$N_{obsunique}$	M_{anom}	-0.100341	0.017338	0.010068
$N_{obsunique}$	T_{anom}	-0.065131	0.123018	0.004242
$N_{obsunique}$	$\Delta I/\sigma I$	-0.077915	0.064922	0.006071
$N_{obsunique}$	$\Delta F/F$	0.090956	0.031089	0.008273

$N_{obsunique}$	f'_{theor}	0.017673	0.675899	0.000312
$N_{obsunique}$	V_{cell}	0.120036	0.004377	0.014409
$N_{obsunique}$	N_{sg}	-0.275638	0	0.075976
$N_{obsunique}$	a	0.081425	0.053704	0.00663
$N_{obsunique}$	b	0.170157	0.00005	0.028953
$N_{obsunique}$	c	0.129025	0.002179	0.016647
$N_{obsunique}$	α	0.061382	0.146147	0.003768
$N_{obsunique}$	β	0.101793	0.015776	0.010362
$N_{obsunique}$	γ	-0.182822	0.000013	0.033424
$N_{obsunique}$	V_s	-0.03354	0.427446	0.001125
$N_{obsunique}$	N_{cell}	0.129106	0.002164	0.016668
$N_{obsunique}$	L	0.026789	0.526234	0.000718
$N_{obsunique}$	V_m	-0.072491	0.085988	0.005255
$N_{obsunique}$	MWS_{ASUVs}	-0.065234	0.122422	0.004256
$N_{obsunique}$	MW_{chain}	0.29067	0	0.084489
$N_{obsunique}$	$N_{atomchain}$	0.286156	0	0.081885
$N_{obsunique}$	N_{molASU}	0.400323	0	0.160259
$N_{obsunique}$	MW_{ASU}	0.584764	0	0.341949
$N_{obsunique}$	$N_{sitesASU}$	0.606049	0	0.367296
$N_{obsunique}$	MWS_{ASU}	-0.058886	0.163292	0.003468
$N_{obsunique}$	I_{ASU}	-0.354903	0	0.125956
$N_{obsunique}$	AV_z	0.029377	0.487033	0.000863
M	T	0.166753	0.000071	0.027807
M	d_{max}	0.305498	0	0.093329
M	d_{min}	0.024924	0.555438	0.000621
M	B	0.155796	0.000209	0.024272
M	d_{inv}	-0.01109	0.793067	0.000123
M	m_{anom}	0.077951	0.064799	0.006076
M	CC_{anom}	0.07128	0.091374	0.005081

M	M_{anom}	0.999032	0	0.998066
M	T_{anom}	0.270732	0	0.073296
M	ΔI/σI	0.110597	0.008688	0.012232
M	ΔF/F	-0.25161	0	0.063308
M	f'_{theor}	-0.127224	0.002514	0.016186
M	V_{cell}	0.33255	0	0.110589
M	N_{sg}	0.492219	0	0.242279
M	a	0.202638	0.000001	0.041062
M	b	0.224775	0	0.050524
M	c	0.204517	0.000001	0.041827
M	α	0.008914	0.833006	0.000079
M	β	-0.157153	0.000184	0.024697
M	γ	0.241474	0	0.05831
M	V_s	0.089529	0.033841	0.008015
M	N_{cell}	0.31625	0	0.100014
M	L	-0.024627	0.560155	0.000606
M	V_m	0.10453	0.013163	0.010926
M	MWS_{ASUVs}	0.038382	0.363761	0.001473
M	MW_{chain}	-0.013897	0.742364	0.000193
M	$N_{atomchain}$	-0.009079	0.829948	0.000082
M	N_{molASU}	-0.10708	0.011081	0.011466
M	MW_{ASU}	-0.084371	0.045579	0.007119
M	$N_{sitesASU}$	-0.129419	0.002111	0.016749
M	MWS_{ASU}	0.050204	0.23473	0.00252
M	I_{ASU}	0.18451	0.000011	0.034044
M	AV_z	-0.018837	0.655883	0.000355
T	d_{max}	0.052198	0.216641	0.002725
T	d_{min}	0.000208	0.996083	0
T	B	0.237291	0	0.056307

T	d_{inv}	-0.022172	0.599923	0.000492
T	m_{anom}	0.016474	0.696764	0.000271
T	CC_{anom}	0.160459	0.000133	0.025747
T	M_{anom}	0.15169	0.000307	0.02301
T	T_{anom}	0.91627	0	0.839551
T	ΔI/σI	0.065607	0.120298	0.004304
T	ΔF/F	0.127357	0.002488	0.01622
T	f'_{theor}	0.198564	0.000002	0.039428
T	V_{cell}	0.122901	0.003521	0.015105
T	N_{sg}	0.223603	0	0.049998
T	a	0.19638	0.000003	0.038565
T	b	0.135561	0.001276	0.018377
T	c	0.074207	0.078798	0.005507
T	α	0.149666	0.000371	0.0224
T	β	0.119754	0.004471	0.014341
T	γ	0.201588	0.000001	0.040638
T	V_s	0.100854	0.016772	0.010172
T	N_{cell}	0.115718	0.006025	0.013391
T	L	-0.057616	0.172574	0.00332
T	V_m	0.10749	0.010774	0.011554
T	MWS_{ASUVs}	-0.031348	0.458277	0.000983
T	MW_{chain}	0.038519	0.362052	0.001484
T	$N_{atomchain}$	0.039749	0.346923	0.00158
T	N_{molASU}	-0.068473	0.104902	0.004688
T	MW_{ASU}	-0.032581	0.440779	0.001062
T	$N_{sitesASU}$	-0.054785	0.194687	0.003001
T	MWS_{ASU}	-0.008932	0.832663	0.00008
T	I_{ASU}	-0.1611	0.000125	0.025953
T	AV_z	0.003269	0.938366	0.000011

d_{max}	d_{min}	0.238624	0	0.056941
d_{max}	B	0.239818	0	0.057513
d_{max}	d_{inv}	-0.154255	0.000242	0.023795
d_{max}	m_{anom}	-0.227894	0	0.051936
d_{max}	CC_{anom}	-0.279757	0	0.078264
d_{max}	M_{anom}	0.303223	0	0.091944
d_{max}	T_{anom}	0.06481	0.12488	0.0042
d_{max}	ΔI/σI	-0.230896	0	0.053313
d_{max}	ΔF/F	-0.092999	0.027486	0.008649
d_{max}	f'_{theor}	-0.155897	0.000207	0.024304
d_{max}	V_{cell}	0.274667	0	0.075442
d_{max}	N_{sg}	0.065751	0.119486	0.004323
d_{max}	a	0.265838	0	0.07067
d_{max}	b	0.320792	0	0.102907
d_{max}	c	0.158885	0.000155	0.025244
d_{max}	α	0.032275	0.445096	0.001042
d_{max}	β	-0.04434	0.294032	0.001966
d_{max}	γ	0.054788	0.194664	0.003002
d_{max}	V_s	0.063551	0.132391	0.004039
d_{max}	N_{cell}	0.270183	0	0.072999
d_{max}	L	-0.016183	0.701859	0.000262
d_{max}	V_m	0.058751	0.164259	0.003452
d_{max}	MWS_{ASUVs}	0.024391	0.56392	0.000595
d_{max}	MW_{chain}	0.270356	0	0.073092
d_{max}	$N_{atomchain}$	0.272707	0	0.074369
d_{max}	N_{molASU}	0.232678	0	0.054139
d_{max}	MW_{ASU}	0.360482	0	0.129947
d_{max}	$N_{sitesASU}$	0.288042	0	0.082968
d_{max}	MWS_{ASU}	0.037945	0.369257	0.00144
d_{max}	I_{ASU}	-0.222251	0	0.049395

d_{max}	AV_z	-0.049444	0.241897	0.002445
d_{min}	B	0.712693	0	0.507931
d_{min}	d_{inv}	-0.89546	0	0.801848
d_{min}	m_{anom}	-0.197555	0.000002	0.039028
d_{min}	CC_{anom}	-0.155283	0.000219	0.024113
d_{min}	M_{anom}	0.035015	0.407388	0.001226
d_{min}	T_{anom}	-0.01012	0.810814	0.000102
d_{min}	ΔI/σI	-0.186242	0.000009	0.034686
d_{min}	ΔF/F	0.168133	0.000062	0.028269
d_{min}	f'_{theor}	-0.058386	0.166898	0.003409
d_{min}	V_{cell}	0.259587	0	0.067386
d_{min}	N_{sg}	0.091779	0.029592	0.008423
d_{min}	a	0.313014	0	0.097978
d_{min}	b	0.320795	0	0.102909
d_{min}	c	0.177204	0.000024	0.031401
d_{min}	α	0.014747	0.727206	0.000217
d_{min}	β	-0.105029	0.01273	0.011031
d_{min}	γ	0.044761	0.289461	0.002004
d_{min}	V_s	0.098065	0.020061	0.009617
d_{min}	N_{cell}	0.256021	0	0.065547
d_{min}	L	-0.019485	0.644851	0.00038
d_{min}	V_m	0.118401	0.004946	0.014019
d_{min}	MWS_{ASUVs}	0.051045	0.226975	0.002606
d_{min}	MW_{chain}	0.186497	0.000009	0.034781
d_{min}	$N_{atomchain}$	0.198642	0.000002	0.039459
d_{min}	N_{molASU}	0.315845	0	0.099758
d_{min}	MW_{ASU}	0.350999	0	0.1232
d_{min}	$N_{sitesASU}$	0.250284	0	0.062642
d_{min}	MWS_{ASU}	0.062357	0.139835	0.003888

d_{min}	I_{ASU}	-0.236572	0	0.055967
d_{min}	AV_z	-0.093395	0.02683	0.008723
B	d_{inv}	-0.630203	0	0.397155
B	m_{anom}	-0.057055	0.176801	0.003255
B	CC_{anom}	0.030042	0.47723	0.000903
B	M_{anom}	0.160948	0.000127	0.025904
B	T_{anom}	0.248517	0	0.061761
B	ΔI/σI	-0.01298	0.758808	0.000168
B	ΔF/F	-0.199803	0.000002	0.039921
B	f'_{theor}	-0.011354	0.788251	0.000129
B	V_{cell}	0.247103	0	0.06106
B	N_{sg}	0.26422	0	0.069812
B	a	0.294362	0	0.086649
B	b	0.295481	0	0.087309
B	c	0.159801	0.000142	0.025536
B	α	-0.082918	0.049447	0.006875
B	β	-0.111136	0.008365	0.012351
B	γ	0.178384	0.000021	0.031821
B	V_s	0.132184	0.001687	0.017473
B	N_{cell}	0.242467	0	0.05879
B	L	-0.113426	0.00711	0.012865
B	V_m	0.148195	0.000424	0.021962
B	MWS_{ASUVs}	0.040149	0.342084	0.001612
B	MW_{chain}	0.083043	0.049106	0.006896
B	$N_{atomchain}$	0.097186	0.021208	0.009445
B	N_{molASU}	0.134501	0.001394	0.018091
B	MW_{ASU}	0.170832	0.000047	0.029183
B	$N_{sitesASU}$	0.062274	0.140363	0.003878
B	MWS_{ASU}	0.057835	0.170951	0.003345

B	I_{ASU}	-0.106191	0.011771	0.011277
B	AV_z	-0.094782	0.02464	0.008984
d_{inv}	m_{anom}	0.150091	0.000356	0.022527
d_{inv}	CC_{anom}	0.120397	0.00426	0.014496
d_{inv}	M_{anom}	-0.020693	0.624478	0.000428
d_{inv}	T_{anom}	-0.00731	0.862727	0.000053
d_{inv}	ΔI/σI	0.156677	0.000192	0.024548
d_{inv}	ΔF/F	-0.183938	0.000011	0.033833
d_{inv}	f'_{theor}	0.031887	0.450579	0.001017
d_{inv}	V_{cell}	-0.238005	0	0.056646
d_{inv}	N_{sg}	-0.095115	0.024138	0.009047
d_{inv}	a	-0.305115	0	0.093095
d_{inv}	b	-0.314627	0	0.09899
d_{inv}	c	-0.168877	0.000057	0.028519
d_{inv}	α	0.01719	0.684268	0.000296
d_{inv}	β	0.062457	0.139195	0.003901
d_{inv}	γ	-0.063424	0.133168	0.004023
d_{inv}	V_s	-0.079407	0.05994	0.006305
d_{inv}	N_{cell}	-0.235043	0	0.055245
d_{inv}	L	0.006382	0.880014	0.000041
d_{inv}	V_m	-0.095678	0.023307	0.009154
d_{inv}	MWS_{ASUVs}	-0.048945	0.246692	0.002396
d_{inv}	MW_{chain}	-0.158109	0.000168	0.024998
d_{inv}	$N_{atomchain}$	-0.166832	0.000071	0.027833
d_{inv}	N_{molASU}	-0.271065	0	0.073476
d_{inv}	MW_{ASU}	-0.29409	0	0.086489
d_{inv}	$N_{sitesASU}$	-0.219255	0	0.048073
d_{inv}	MWS_{ASU}	-0.056065	0.184445	0.003143
d_{inv}	I_{ASU}	0.288625	0	0.083304

d_{inv}	AV_z	0.067755	0.108605	0.004591
m_{anom}	CC_{anom}	0.800133	0	0.640213
m_{anom}	M_{anom}	0.080777	0.055643	0.006525
m_{anom}	T_{anom}	0.11594	0.005929	0.013442
m_{anom}	ΔI/σI	0.908074	0	0.824598
m_{anom}	ΔF/F	0.019654	0.641976	0.000386
m_{anom}	f'_{theor}	0.140219	0.000859	0.019661
m_{anom}	V_{cell}	-0.065598	0.120347	0.004303
m_{anom}	N_{sg}	0.212855	0	0.045307
m_{anom}	a	-0.091723	0.029692	0.008413
m_{anom}	b	-0.040559	0.337167	0.001645
m_{anom}	c	0.002067	0.961014	0.000004
m_{anom}	α	-0.165057	0.000085	0.027244
m_{anom}	β	-0.106245	0.011727	0.011288
m_{anom}	γ	0.09023	0.032465	0.008141
m_{anom}	V_s	-0.061657	0.144342	0.003802
m_{anom}	N_{cell}	-0.062861	0.136653	0.003952
m_{anom}	L	-0.065829	0.119042	0.004334
m_{anom}	V_m	-0.05219	0.216709	0.002724
m_{anom}	MWS_{ASUVs}	-0.0887	0.035533	0.007868
m_{anom}	MW_{chain}	-0.109288	0.009519	0.011944
m_{anom}	$N_{atomchain}$	-0.115287	0.006217	0.013291
m_{anom}	N_{molASU}	-0.187929	0.000007	0.035317
m_{anom}	MW_{ASU}	-0.212722	0	0.04525
m_{anom}	$N_{sitesASU}$	-0.154886	0.000228	0.02399
m_{anom}	MWS_{ASU}	-0.111186	0.008336	0.012362
m_{anom}	I_{ASU}	0.235855	0	0.055627
m_{anom}	AV_z	0.066551	0.115042	0.004429

CC_{anom}	M_{anom}	0.07226	0.086995	0.005222
CC_{anom}	T_{anom}	0.229073	0	0.052474
CC_{anom}	ΔI/σI	0.840959	0	0.707212
CC_{anom}	ΔF/F	0.028364	0.502194	0.000804
CC_{anom}	f'_{theor}	0.190764	0.000005	0.036391
CC_{anom}	V_{cell}	-0.061816	0.143306	0.003821
CC_{anom}	N_{sg}	0.252862	0	0.063939
CC_{anom}	a	-0.071869	0.088722	0.005165
CC_{anom}	b	-0.040514	0.337713	0.001641
CC_{anom}	c	-0.006942	0.869573	0.000048
CC_{anom}	α	-0.053495	0.205415	0.002862
CC_{anom}	β	-0.073704	0.080852	0.005432
CC_{anom}	γ	0.163337	0.0001	0.026679
CC_{anom}	V_s	-0.02532	0.549163	0.000641
CC_{anom}	N_{cell}	-0.060561	0.151626	0.003668
CC_{anom}	L	-0.057209	0.175637	0.003273
CC_{anom}	V_m	-0.009174	0.828212	0.000084
CC_{anom}	MWS_{ASUVs}	-0.073263	0.082692	0.005367
CC_{anom}	MW_{chain}	-0.136024	0.001227	0.018503
CC_{anom}	$N_{atomchain}$	-0.142805	0.000686	0.020393
CC_{anom}	N_{molASU}	-0.190402	0.000005	0.036253
CC_{anom}	MW_{ASU}	-0.235652	0	0.055532
CC_{anom}	$N_{sitesASU}$	-0.190411	0.000005	0.036256
CC_{anom}	MWS_{ASU}	-0.085325	0.043183	0.00728
CC_{anom}	I_{ASU}	0.218658	0	0.047811
CC_{anom}	AV_z	0.073283	0.082607	0.00537
M_{anom}	T_{anom}	0.253586	0	0.064306
M_{anom}	ΔI/σI	0.113366	0.007141	0.012852
M_{anom}	ΔF/F	-0.253977	0	0.064504

M_{anom}	f'_{theor}	-0.134308	0.001416	0.018039
M_{anom}	V_{cell}	0.330603	0	0.109298
M_{anom}	N_{sg}	0.497825	0	0.24783
M_{anom}	a	0.196693	0.000003	0.038688
M_{anom}	b	0.218902	0	0.047918
M_{anom}	c	0.210497	0	0.044309
M_{anom}	α	0.007421	0.860651	0.000055
M_{anom}	β	-0.164615	0.000088	0.027098
M_{anom}	γ	0.241017	0	0.058089
M_{anom}	V_s	0.085216	0.04345	0.007262
M_{anom}	N_{cell}	0.314442	0	0.098874
M_{anom}	L	-0.02469	0.559153	0.00061
M_{anom}	V_m	0.101309	0.016283	0.010263
M_{anom}	MWS_{ASUVs}	0.042867	0.310375	0.001838
M_{anom}	MW_{chain}	-0.021568	0.609895	0.000465
M_{anom}	$N_{atomchain}$	-0.017253	0.683174	0.000298
M_{anom}	N_{molASU}	-0.110011	0.009052	0.012102
M_{anom}	MW_{ASU}	-0.092449	0.028419	0.008547
M_{anom}	$N_{sitesASU}$	-0.136137	0.001215	0.018533
M_{anom}	MWS_{ASU}	0.053101	0.208781	0.00282
M_{anom}	I_{ASU}	0.198424	0.000002	0.039372
M_{anom}	AV_z	-0.016574	0.69501	0.000275
T_{anom}	ΔI/σI	0.149563	0.000374	0.022369
T_{anom}	ΔF/F	0.036982	0.381539	0.001368
T_{anom}	f'_{theor}	0.172731	0.000038	0.029836
T_{anom}	V_{cell}	0.146609	0.000489	0.021494
T_{anom}	N_{sg}	0.300844	0	0.090507
T_{anom}	a	0.198065	0.000002	0.03923
T_{anom}	b	0.149603	0.000373	0.022381

T_{anom}	c	0.118616	0.004868	0.01407
T_{anom}	α	0.145874	0.000522	0.021279
T_{anom}	β	0.08812	0.03676	0.007765
T_{anom}	γ	0.260381	0	0.067798
T_{anom}	V_s	0.110153	0.008963	0.012134
T_{anom}	N_{cell}	0.138585	0.000988	0.019206
T_{anom}	L	-0.062278	0.140339	0.003878
T_{anom}	V_m	0.121616	0.003884	0.01479
T_{anom}	MWS_{ASUVs}	-0.038524	0.361995	0.001484
T_{anom}	MW_{chain}	0.030034	0.477342	0.000902
T_{anom}	$N_{atomchain}$	0.031695	0.453323	0.001005
T_{anom}	N_{molASU}	-0.148821	0.0004	0.022148
T_{anom}	MW_{ASU}	-0.095676	0.023311	0.009154
T_{anom}	$N_{sitesASU}$	-0.14072	0.000822	0.019802
T_{anom}	MWS_{ASU}	-0.013983	0.74082	0.000196
T_{anom}	I_{ASU}	-0.060139	0.154501	0.003617
T_{anom}	AV_z	-0.001143	0.978423	0.000001
ΔI/σI	ΔF/F	0.007089	0.866838	0.00005
ΔI/σI	f'_{theor}	0.121395	0.00395	0.014737
ΔI/σI	V_{cell}	-0.076729	0.069122	0.005887
ΔI/σI	N_{sg}	0.258124	0	0.066628
ΔI/σI	a	-0.08895	0.035015	0.007912
ΔI/σI	b	-0.082709	0.050027	0.006841
ΔI/σI	c	-0.006849	0.871303	0.000047
ΔI/σI	α	-0.07934	0.060155	0.006295
ΔI/σI	β	-0.092786	0.027844	0.008609
ΔI/σI	γ	0.14307	0.00067	0.020469
ΔI/σI	V_s	-0.04011	0.34255	0.001609
ΔI/σI	N_{cell}	-0.076706	0.069209	0.005884

ΔI/σI	L	-0.041574	0.325213	0.001728
ΔI/σI	V_m	-0.027889	0.509377	0.000778
ΔI/σI	MWS_{ASUVs}	-0.098651	0.019327	0.009732
ΔI/σI	MW_{chain}	-0.163314	0.000101	0.026672
ΔI/σI	$N_{atomchain}$	-0.168819	0.000058	0.0285
ΔI/σI	N_{molASU}	-0.186158	0.000009	0.034655
ΔI/σI	MW_{ASU}	-0.23311	0	0.05434
ΔI/σI	$N_{sitesASU}$	-0.16585	0.000078	0.027506
ΔI/σI	MWS_{ASU}	-0.117513	0.005282	0.013809
ΔI/σI	I_{ASU}	0.310995	0	0.096718
ΔI/σI	AV_z	0.063918	0.130165	0.004086
ΔF/F	f'_{theor}	0.154232	0.000242	0.023787
ΔF/F	V_{cell}	0.012174	0.773376	0.000148
ΔF/F	N_{sg}	-0.268056	0	0.071854
ΔF/F	a	-0.007734	0.854852	0.00006
ΔF/F	b	0.041687	0.323901	0.001738
ΔF/F	c	0.085014	0.043951	0.007227
ΔF/F	α	0.242717	0	0.058912
ΔF/F	β	0.069695	0.09883	0.004857
ΔF/F	γ	-0.196271	0.000003	0.038522
ΔF/F	V_s	-0.037428	0.37582	0.001401
ΔF/F	N_{cell}	0.011349	0.788351	0.000129
ΔF/F	L	-0.039192	0.353727	0.001536
ΔF/F	V_m	-0.050528	0.231717	0.002553
ΔF/F	MWS_{ASUVs}	-0.044446	0.292872	0.001975
ΔF/F	MW_{chain}	0.241983	0	0.058556
ΔF/F	$N_{atomchain}$	0.236739	0	0.056045
ΔF/F	N_{molASU}	0.209418	0.000001	0.043856
ΔF/F	MW_{ASU}	0.28788	0	0.082875

ΔF/F	$N_{sitesASU}$	0.297038	0	0.088231
ΔF/F	MWS_{ASU}	-0.0412	0.329587	0.001697
ΔF/F	I_{ASU}	-0.478383	0	0.228851
ΔF/F	AV_z	0.010913	0.796305	0.000119
f'_{theor}	V_{cell}	-0.009302	0.825845	0.000087
f'_{theor}	N_{sg}	-0.03976	0.346783	0.001581
f'_{theor}	a	0.047069	0.265293	0.002215
f'_{theor}	b	0.008539	0.839931	0.000073
f'_{theor}	c	-0.074957	0.075813	0.005619
f'_{theor}	α	0.036867	0.383018	0.001359
f'_{theor}	β	0.072591	0.085556	0.005269
f'_{theor}	γ	-0.007523	0.858759	0.000057
f'_{theor}	V_s	0.067058	0.112295	0.004497
f'_{theor}	N_{cell}	-0.0082	0.846196	0.000067
f'_{theor}	L	0.025183	0.551326	0.000634
f'_{theor}	V_m	0.061181	0.147471	0.003743
f'_{theor}	MWS_{ASUVs}	0.015304	0.717344	0.000234
f'_{theor}	MW_{chain}	0.03384	0.423315	0.001145
f'_{theor}	$N_{atomchain}$	0.036793	0.383979	0.001354
f'_{theor}	N_{molASU}	-0.011001	0.794694	0.000121
f'_{theor}	MW_{ASU}	0.004696	0.911545	0.000022
f'_{theor}	$N_{sitesASU}$	-0.003745	0.929412	0.000014
f'_{theor}	MWS_{ASU}	0.03332	0.430491	0.00111
f'_{theor}	I_{ASU}	-0.125508	0.002878	0.015752
f'_{theor}	AV_z	-0.013218	0.754536	0.000175
V_{cell}	N_{sg}	0.241783	0	0.058459
V_{cell}	a	0.545946	0	0.298057
V_{cell}	b	0.571229	0	0.326303

V_{cell}	c	0.447857	0	0.200576
V_{cell}	α	0.01966	0.641883	0.000386
V_{cell}	β	-0.073708	0.080837	0.005433
V_{cell}	γ	0.040894	0.333193	0.001672
V_{cell}	V_s	0.063002	0.135776	0.003969
V_{cell}	N_{cell}	0.98942	0	0.978952
V_{cell}	L	-0.021566	0.609926	0.000465
V_{cell}	V_m	0.056692	0.179577	0.003214
V_{cell}	MWS_{ASUVs}	-0.019675	0.64162	0.000387
V_{cell}	MW_{chain}	0.257373	0	0.066241
V_{cell}	$N_{atomchain}$	0.250397	0	0.062699
V_{cell}	N_{molASU}	0.229531	0	0.052684
V_{cell}	MW_{ASU}	0.363624	0	0.132223
V_{cell}	$N_{sitesASU}$	0.293865	0	0.086357
V_{cell}	MWS_{ASU}	-0.00729	0.863085	0.000053
V_{cell}	I_{ASU}	-0.209725	0.000001	0.043985
V_{cell}	AV_z	0.044102	0.296633	0.001945
N_{sg}	a	0.258303	0	0.06672
N_{sg}	b	0.172685	0.000039	0.02982
N_{sg}	c	0.235977	0	0.055685
N_{sg}	α	0.014151	0.737821	0.0002
N_{sg}	β	-0.299188	0	0.089513
N_{sg}	γ	0.788098	0	0.621098
N_{sg}	V_s	0.149871	0.000364	0.022461
N_{sg}	N_{cell}	0.223436	0	0.049924
N_{sg}	L	-0.041302	0.328393	0.001706
N_{sg}	V_m	0.194975	0.000003	0.038015
N_{sg}	MWS_{ASUVs}	0.012842	0.761297	0.000165
N_{sg}	MW_{chain}	-0.14786	0.000437	0.021862

N_{sg}	$N_{atomchain}$	-0.155773	0.00021	0.024265
N_{sg}	N_{molASU}	-0.254922	0	0.064985
N_{sg}	MW_{ASU}	-0.281318	0	0.07914
N_{sg}	$N_{sitesASU}$	-0.272	0	0.073984
N_{sg}	MWS_{ASU}	0.020569	0.626554	0.000423
N_{sg}	I_{ASU}	0.378535	0	0.143289
N_{sg}	AV_z	0.069301	0.100755	0.004803
a	b	0.45929	0	0.210947
a	c	0.022581	0.593212	0.00051
a	α	0.028085	0.506397	0.000789
a	β	0.09411	0.02568	0.008857
a	γ	0.190354	0.000006	0.036235
a	V_s	0.088	0.037017	0.007744
a	N_{cell}	0.535843	0	0.287128
a	L	-0.023075	0.585146	0.000532
a	V_m	0.081339	0.053958	0.006616
a	MWS_{ASUVs}	-0.039856	0.345616	0.001589
a	MW_{chain}	0.13004	0.002008	0.01691
a	$N_{atomchain}$	0.122915	0.003517	0.015108
a	N_{molASU}	0.368116	0	0.135509
a	MW_{ASU}	0.368248	0	0.135607
a	$N_{sitesASU}$	0.347348	0	0.12065
a	MWS_{ASU}	-0.026693	0.527719	0.000712
a	I_{ASU}	-0.228881	0	0.052386
a	AV_z	0.051902	0.219255	0.002694
b	c	0.030263	0.473993	0.000916
b	α	0.059638	0.157971	0.003557
b	β	-0.077287	0.067121	0.005973

b	γ	0.116371	0.005745	0.013542
b	V_s	0.126579	0.002646	0.016022
b	N_{cell}	0.556852	0	0.310084
b	L	-0.033652	0.425904	0.001132
b	V_m	0.110849	0.008536	0.012287
b	MWS_{ASUVs}	0.020814	0.622456	0.000433
b	MW_{chain}	0.300809	0	0.090486
b	$N_{atomchain}$	0.296805	0	0.088093
b	N_{molASU}	0.233494	0	0.054519
b	MW_{ASU}	0.385423	0	0.148551
b	$N_{sitesASU}$	0.285058	0	0.081258
b	MWS_{ASU}	0.046933	0.266675	0.002203
b	I_{ASU}	-0.313664	0	0.098385
b	AV_z	0.02524	0.550428	0.000637
c	α	0.043707	0.300981	0.00191
c	β	-0.173822	0.000034	0.030214
c	γ	0.104834	0.012898	0.01099
c	V_s	0.122232	0.003706	0.014941
c	N_{cell}	0.440174	0	0.193753
c	L	-0.031297	0.45901	0.00098
c	V_m	0.123016	0.00349	0.015133
c	MWS_{ASUVs}	-0.028376	0.502008	0.000805
c	MW_{chain}	0.309184	0	0.095595
c	$N_{atomchain}$	0.301647	0	0.090991
c	N_{molASU}	0.08724	0.038688	0.007611
c	MW_{ASU}	0.281907	0	0.079472
c	$N_{sitesASU}$	0.236133	0	0.055759
c	MWS_{ASU}	-0.004259	0.919758	0.000018
c	I_{ASU}	-0.193271	0.000004	0.037354

c	AV_z	0.039555	0.349279	0.001565
α	β	0.262442	0	0.068876
α	γ	0.144278	0.000602	0.020816
α	V_s	0.024854	0.556552	0.000618
α	N_{cell}	0.006533	0.877193	0.000043
α	L	0.149864	0.000364	0.022459
α	V_m	0.02947	0.485664	0.000868
α	MWS_{ASUVs}	0.031646	0.454013	0.001001
α	MW_{chain}	0.051479	0.223038	0.00265
α	$N_{atomchain}$	0.012685	0.764128	0.000161
α	N_{molASU}	0.04101	0.331825	0.001682
α	MW_{ASU}	0.095668	0.023323	0.009152
α	$N_{sitesASU}$	0.07754	0.066226	0.006013
α	MWS_{ASU}	0.037851	0.370446	0.001433
α	I_{ASU}	-0.070104	0.09686	0.004915
α	AV_z	0.204286	0.000001	0.041733
β	γ	-0.101749	0.015821	0.010353
β	V_s	-0.070717	0.093969	0.005001
β	N_{cell}	-0.067338	0.110801	0.004534
β	L	0.119587	0.004527	0.014301
β	V_m	-0.088173	0.036646	0.007774
β	MWS_{ASUVs}	0.010238	0.808654	0.000105
β	MW_{chain}	-0.043646	0.301657	0.001905
β	$N_{atomchain}$	-0.04607	0.275574	0.002122
β	N_{molASU}	0.121619	0.003883	0.014791
β	MW_{ASU}	0.076998	0.068151	0.005929
β	$N_{sitesASU}$	0.068162	0.106489	0.004646
β	MWS_{ASU}	0.00429	0.919174	0.000018

β	I_{ASU}	-0.162619	0.000108	0.026445
β	AV_z	0.01949	0.644757	0.00038
γ	V_s	0.120902	0.004101	0.014617
γ	N_{cell}	0.032609	0.440395	0.001063
γ	L	-0.008805	0.83501	0.000078
γ	V_m	0.156946	0.000187	0.024632
γ	MWS_{ASUVs}	0.027395	0.516915	0.00075
γ	MW_{chain}	-0.07815	0.064115	0.006107
γ	$N_{atomchain}$	-0.089577	0.033745	0.008024
γ	N_{molASU}	-0.190764	0.000005	0.036391
γ	MW_{ASU}	-0.177849	0.000022	0.03163
γ	$N_{sitesASU}$	-0.181147	0.000016	0.032814
γ	MWS_{ASU}	0.035345	0.402979	0.001249
γ	I_{ASU}	0.262256	0	0.068778
γ	AV_z	0.079655	0.05914	0.006345
V_s	N_{cell}	-0.026379	0.532583	0.000696
V_s	L	0.010444	0.804868	0.000109
V_s	V_m	0.981723	0	0.96378
V_s	MWS_{ASUVs}	-0.218282	0	0.047647
V_s	MW_{chain}	0.188559	0.000007	0.035555
V_s	$N_{atomchain}$	0.190301	0.000006	0.036214
V_s	N_{molASU}	-0.184098	0.000011	0.033892
V_s	MW_{ASU}	-0.102866	0.014702	0.010581
V_s	$N_{sitesASU}$	-0.090073	0.032768	0.008113
V_s	MWS_{ASU}	-0.011714	0.781708	0.000137
V_s	I_{ASU}	-0.006824	0.87176	0.000047
V_s	AV_z	-0.018585	0.66019	0.000345

N_{cell}	L	-0.021441	0.611998	0.00046
N_{cell}	V_m	-0.033625	0.426276	0.001131
N_{cell}	MWS_{ASUVs}	-0.000396	0.992525	0
N_{cell}	MW_{chain}	0.227687	0	0.051841
N_{cell}	$N_{atomchain}$	0.229467	0	0.052655
N_{cell}	N_{molASU}	0.268045	0	0.071848
N_{cell}	MW_{ASU}	0.385302	0	0.148457
N_{cell}	$N_{sitesASU}$	0.314736	0	0.099059
N_{cell}	MWS_{ASU}	-0.006158	0.884193	0.000038
N_{cell}	I_{ASU}	-0.21786	0	0.047463
N_{cell}	AV_z	-0.003262	0.93849	0.000011
L	V_m	0.0041	0.922748	0.000017
L	MWS_{ASUVs}	-0.003895	0.926585	0.000015
L	MW_{chain}	-0.046772	0.26832	0.002188
L	$N_{atomchain}$	-0.046885	0.267166	0.002198
L	N_{molASU}	0.089477	0.033945	0.008006
L	MW_{ASU}	0.017661	0.676099	0.000312
L	$N_{sitesASU}$	0.006967	0.869105	0.000049
L	MWS_{ASU}	-0.000601	0.988654	0
L	I_{ASU}	-0.021659	0.608386	0.000469
L	AV_z	0.002224	0.958053	0.000005
V_m	MWS_{ASUVs}	-0.203766	0.000001	0.041521
V_m	MW_{chain}	0.201296	0.000002	0.04052
V_m	$N_{atomchain}$	0.201937	0.000001	0.040779
V_m	N_{molASU}	-0.224485	0	0.050393
V_m	MW_{ASU}	-0.138245	0.001017	0.019112
V_m	$N_{sitesASU}$	-0.127118	0.002536	0.016159
V_m	MWS_{ASU}	-0.008994	0.831529	0.000081

V_m	I_{ASU}	0.041175	0.329879	0.001695
V_m	AV_z	-0.010268	0.808101	0.000105
MWS_{ASUVs}	MW_{chain}	-0.038341	0.36428	0.00147
MWS_{ASUVs}	$N_{atomchain}$	-0.042745	0.311761	0.001827
MWS_{ASUVs}	N_{molASU}	0.025671	0.543645	0.000659
MWS_{ASUVs}	MW_{ASU}	0.02911	0.491013	0.000847
MWS_{ASUVs}	$N_{sitesASU}$	-0.212131	0	0.045
MWS_{ASUVs}	MWS_{ASU}	0.972152	0	0.94508
MWS_{ASUVs}	I_{ASU}	-0.008908	0.833121	0.000079
MWS_{ASUVs}	AV_z	0.028064	0.506728	0.000788
MW_{chain}	$N_{atomchain}$	0.985546	0	0.971301
MW_{chain}	N_{molASU}	-0.149828	0.000365	0.022448
MW_{chain}	MW_{ASU}	0.412332	0	0.170018
MW_{chain}	$N_{sitesASU}$	0.300168	0	0.090101
MW_{chain}	MWS_{ASU}	-0.001508	0.971542	0.000002
MW_{chain}	I_{ASU}	-0.462634	0	0.21403
MW_{chain}	AV_z	0.065703	0.119755	0.004317
$N_{atomchain}$	N_{molASU}	-0.118776	0.00481	0.014108
$N_{atomchain}$	MW_{ASU}	0.419023	0	0.17558
$N_{atomchain}$	$N_{sitesASU}$	0.311617	0	0.097105
$N_{atomchain}$	MWS_{ASU}	-0.005414	0.898095	0.000029
$N_{atomchain}$	I_{ASU}	-0.466452	0	0.217577
$N_{atomchain}$	AV_z	-0.096753	0.021793	0.009361
N_{molASU}	MW_{ASU}	0.737891	0	0.544483
N_{molASU}	$N_{sitesASU}$	0.734071	0	0.53886
N_{molASU}	MWS_{ASU}	0.002663	0.949769	0.000007

N_{molASU}	I_{ASU}	-0.395268	0	0.156237
N_{molASU}	AV_z	-0.167278	0.000068	0.027982
MW_{ASU}	$N_{sitesASU}$	0.888157	0	0.788822
MW_{ASU}	MWS_{ASU}	0.019884	0.638091	0.000395
MW_{ASU}	I_{ASU}	-0.516341	0	0.266608
MW_{ASU}	AV_z	-0.041099	0.330776	0.001689
$N_{sitesASU}$	MWS_{ASU}	-0.223483	0	0.049945
$N_{sitesASU}$	I_{ASU}	-0.44862	0	0.20126
$N_{sitesASU}$	AV_z	-0.066459	0.115544	0.004417
MWS_{ASU}	I_{ASU}	-0.025491	0.546472	0.00065
MWS_{ASU}	AV_z	0.021793	0.606167	0.000475
I_{ASU}	AV_z	0.048071	0.255243	0.002311

Table S4 Highest scoring features found for different classifiers in the initial training step using the full feature set.

Linear SVM	Decision tree	Decision tree with Bagging	Decision tree with AdaBoost	Random forest	Extreme randomized forest
I/σ	CC_{anom}	m_{anom}	CC_{anom}	m_{anom}	CC_{anom}
CC_{anom}	ΔF/F	CC_{anom}	m_{anom}	CC_{anom}	Δ I/σI
m_{anom}	$CC_{1/2}$	Δ I/σI	d_{max}	Δ I/σI	f''_{theor}
$CC_{1/2}$	d_{max}	f''_{theor}	f''_{theor}	f''_{theor}	m_{anom}
ΔI/σI	m_{anom}	d_{max}	$CC_{1/2}$	d_{max}	d_{max}
d_{max}	$R_{p.i.m.}$(I+/I-)	ΔF/F	N_{sg}	ΔF/F	ΔF/F
	f''_{theor}		$N_{obstotal}$		T_{anom}
			$N_{atomchain}$		MW_{ASU}
			Δ I/σI		T

Table S5 Scores for the classification outcomes and performance metrics found for different classifiers in the initial training step using the full feature set.

	SVM linear kernel	SVM rbf kernel	Decision tree	Decision tree with Bagging	Decision tree with AdaBoost	Random forest	Extreme randomized forest
ACC (%)	95	96	88	94	98	94	94
Class Error (%)	5	4	12	6	2	6	6
Sensitivity (%)	94	96	88	96	97	96	90
Specificity (%)	98	96	87	91	100	91	100
FPR (%)	2	4	13	9	0	9	0
Precision (%)	99	98	93	96	100	96	100
F1 score (%)	96	97	91	96	98	96	95
ROC AUC (%)	97	99	89	99	99	99	98
TP	88	90	83	90	91	90	85
TN	46	45	41	43	47	43	47
FP	1	2	6	4	0	4	0
FN	6	4	11	4	3	4	9

Table S6 Scores for the classification outcomes and performance metrics found for different classifiers after retraining using the small feature set (CC_{anom}, $\Delta I/\sigma I$, m_{anom}, d_{max}, $\Delta F/F$, f''_{theor}).

	SVM linear kernel	SVM rbf kernel	Decision tree	Decision tree with Bagging	Decision tree with AdaBoost	Random forest	Extreme randomized forest
ACC (%)	91	92	91	94	95	94	89
Class Error (%)	9	8	9	6	5	6	11
Sensitivity (%)	94	96	91	95	96	95	95
Specificity (%)	85	85	89	91	94	91	79
FPR (%)	15	15	11	9	6	9	21
Precision (%)	93	93	95	96	97	96	90
F1 score (%)	93	94	93	95	96	95	92
ROC AUC (%)	98	98	93	99	99	99	97
TP	88	90	86	89	90	89	89
TN	40	40	42	43	44	43	37
FP	7	7	5	4	3	4	10
FN	6	4	8	5	4	5	5

Figure S1 Schema of how to train and assess a set of classifiers.

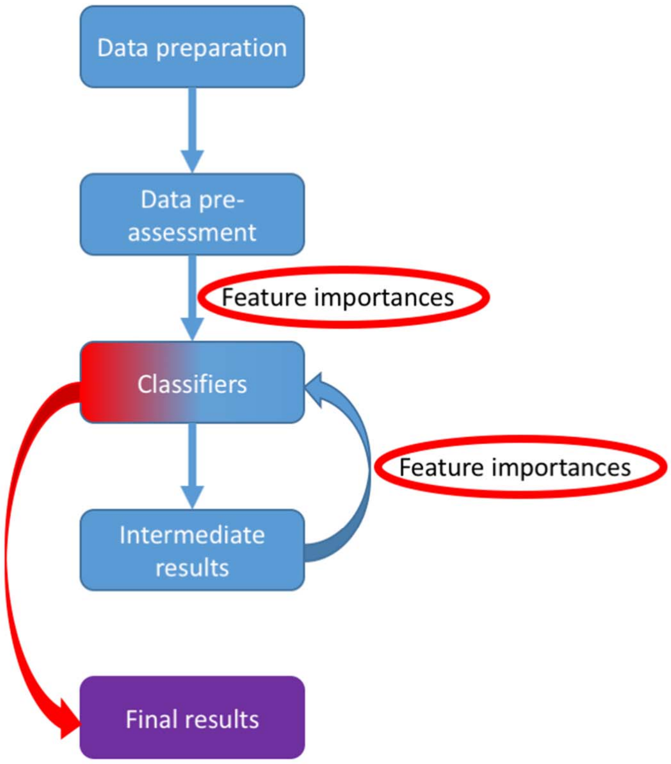

Supplement: Supplementary file 1 [file m-07-00342-sup1.pdf]
